# Longitudinal dynamics and cross-domain interactions of eukaryotic populations in wastewater treatment plants

**DOI:** 10.1093/ismejo/wraf058

**Published:** 2025-04-04

**Authors:** Yue Huang, Xuemei Mao, Xiawan Zheng, Yuxiang Zhao, Dou Wang, Mengying Wang, Yiqiang Chen, Lei Liu, Yulin Wang, Martin F Polz, Tong Zhang

**Affiliations:** Environmental Microbiome Engineering and Biotechnology Laboratory, Center for Environmental Engineering Research, Department of Civil Engineering, The University of Hong Kong, Hong Kong SAR, 999077, China; Environmental Microbiome Engineering and Biotechnology Laboratory, Center for Environmental Engineering Research, Department of Civil Engineering, The University of Hong Kong, Hong Kong SAR, 999077, China; Environmental Microbiome Engineering and Biotechnology Laboratory, Center for Environmental Engineering Research, Department of Civil Engineering, The University of Hong Kong, Hong Kong SAR, 999077, China; Environmental Microbiome Engineering and Biotechnology Laboratory, Center for Environmental Engineering Research, Department of Civil Engineering, The University of Hong Kong, Hong Kong SAR, 999077, China; Environmental Microbiome Engineering and Biotechnology Laboratory, Center for Environmental Engineering Research, Department of Civil Engineering, The University of Hong Kong, Hong Kong SAR, 999077, China; Environmental Microbiome Engineering and Biotechnology Laboratory, Center for Environmental Engineering Research, Department of Civil Engineering, The University of Hong Kong, Hong Kong SAR, 999077, China; Environmental Microbiome Engineering and Biotechnology Laboratory, Center for Environmental Engineering Research, Department of Civil Engineering, The University of Hong Kong, Hong Kong SAR, 999077, China; Environmental Microbiome Engineering and Biotechnology Laboratory, Center for Environmental Engineering Research, Department of Civil Engineering, The University of Hong Kong, Hong Kong SAR, 999077, China; Environmental Microbiome Engineering and Biotechnology Laboratory, Center for Environmental Engineering Research, Department of Civil Engineering, The University of Hong Kong, Hong Kong SAR, 999077, China; Division of Microbial Ecology, Centre for Microbiology and Environmental Systems Science, University of Vienna, Vienna, 1030, Austria; Environmental Microbiome Engineering and Biotechnology Laboratory, Center for Environmental Engineering Research, Department of Civil Engineering, The University of Hong Kong, Hong Kong SAR, 999077, China; School of Public Health, The University of Hong Kong, Hong Kong SAR, 999077, China; Macau Institute for Applied Research in Medicine and Health, Macau University of Science and Technology, Macao SAR, 999078, China; State Key Laboratory of Marine Pollution, City University of Hong Kong, Hong Kong SAR, 999077, China; Shenzhen Innovation and Research Institute, The University of Hong Kong, Shenzhen, 518057, China

**Keywords:** eukaryotes, activated sludge, wastewater treatment plants, cross-domain interactions, metatranscriptomics

## Abstract

Activated sludge is a large reservoir of novel microorganisms and microbial genetic diversity. While much attention has been given to the profile and functions of prokaryotes, the eukaryotic diversity remains largely unexplored. In this study, we analysed longitudinal activated sludge samples spanning 13 years from the largest secondary wastewater treatment plants in Hong Kong, unveiling a wealth of eukaryotic taxa and 681 856 non-redundant protein-coding genes, the majority (416 044) of which appeared novel. Ciliophora was the most dominant phylum with a significant increase after a transient intervention (bleaching event). Our metagenomic analysis reveals close linkage and covariation of eukaryotes, prokaryotes, and prokaryotic viruses (phages), indicating common responses to environmental changes such as transient intervention and intermittent fluctuations. Furthermore, high-resolution cross-domain relationships were interpreted by S-map, demonstrating a predatory role of Arthropoda, Ascomycota, Mucoromycota, and Rotifera. This high-resolution profile of microbial dynamics expands our knowledge on yet-to-be-cultured populations and their cross-domain interactions and highlights the ecological importance of eukaryotes in the activated sludge ecosystem.

## Introduction

Biological wastewater treatment plants (WWTPs) are the most important manmade ecosystems worldwide, being tightly linked to our living conditions and ecosystem health. Deep investigations of microbial diversity within WWTPs have shown that they represent a unique inventory of novel microorganisms and microbial genetic diversity. Determining the community structure and potential genetic functions in full-scale WWTPs is critical to tracking treatment efficiency and carrying out microbial resource management [1]. However, while previous analyses have primarily focused on taxonomic profiles and functions of bacteria and archaea in activated sludge [2, 3], our knowledge of eukaryotes in WWTPs lags significantly behind that of prokaryotes.

Although eukaryotic populations only make up a small proportion of total microorganisms in activated sludge, they have been shown to contribute to vital clean-up processes, such as organic matter elimination [4], and, through their predatory activity, they can remove pathogens [5] and structure the microbial community. Previous studies focused on culturing and microscopic analysis, providing visual evidence for the presence of eukaryotes and their predatory activities on bacteria [6, 7]. Protists are the most highly represented eukaryotes in activated sludge, making up 5% to 12% of the dry weight [8, 9]. Curds and Hawkes [8] microscopically identified over 200 species of protists with ciliates accounting for 70% of species. This is only a small fraction of the thousands of protists present in this ecosystem. Assessing populations of protists, such as ciliates and flagellates, has proven useful as bioindicators of effluent quality [10–12] and has been used for WWTP management. However, culture-dependent and microscopic methods fail to comprehensively address eukaryotic diversity, necessitating culture-independent approaches.

High-throughput sequencing technologies have significantly advanced our understanding of the composition and diversity of environmental microorganisms. Marker-gene-based analyses of activated sludge communities, focusing on sequencing of 18S ribosomal RNA (rRNA) genes and internal transcribed spacer regions, have uncovered thousands of eukaryotic operational taxonomic units that are dominated by ciliates, arthropods, fungi, and rotifers [4, 13]. However, such maker gene-based methods have inherent limitations. They obscure functional diversity and are, especially for eukaryotes, heavily biased due to 18S rRNA gene copy number variation, which can range from a few to hundreds of copies in a single genome [14, 15]. This makes quantitative analysis based on relative gene abundances challenging. Although technological advances in the past decade have improved our ability to interpret eukaryotic data directly from metagenomic data, this approach is lagging far behind cultivation-based methods in constructing eukaryotic genomes, which contrasts with the other two domains of life. Eukaryotic signals are frequently overwhelmed by the abundance of prokaryotes or filtered out due to inadequate sequencing depth and stringent quality controls. The larger and more complex genomes of eukaryotes further complicate genome reconstruction and characterization, limiting our ability to fully explore their diversity and ecological roles in activated sludge ecosystems.

We took advantage of metagenomic data sets obtained in our previous studies [16–18] and sequenced additional longitudinal samples to collect a 13-year-long metagenomic sequence dataset of activated sludge in the largest secondary WWTPs in Hong Kong. Our goal is to construct a comprehensive taxonomy and gene catalog for eukaryotes in activated sludge ecosystems, laying the groundwork for a deeper understanding of their ecological and biochemical roles. We investigated temporal patterns in relation to environmental conditions. In addition, a regularized sequential locally weighted global linear map (S-map) method was applied to explore the across-domain microbial interactions, which is a standard empirical dynamic modeling approach to characterize the varying patterns of microbial interaction in complex ecosystems from the community time series profile [19, 20]. These analyses advance our understanding of the composition and function of eukaryotes in activated sludge, offering new insights that can enhance the management of WWTPs.

## Materials and methods

### Sampling campaign

Briefly, 143 longitudinal aerobic activated sludge samples were collected monthly over 13 years (from June 2007 to December 2019) from the Sha Tin (ST) Sewage Treatment Works (22.406 N, 114.213 E), which is the largest secondary WWTPs in Hong Kong with 260 000 m^3^ wastewater treatment capacity, serving for over 650 000 citizens. The bleaching solution (sodium hypochlorite) was added to mitigate the seasonal foaming problem in December 2009. Small trial experiments showed an efficient alleviation of biological foaming by the addition of bleach solution, and full trial experiments were performed subsequently in months with bleaching events and stopped in 2014. The detailed description can be found in our previous study [16]. To investigate the biogeographic distribution patterns of eukaryotes, one-year activated sludge samples were collected monthly from two more local municipal WWTPs (Shek Wu Hui (SWH) and Stanley (STL) WWTPs) from January 2018 to January 2019. In addition, three more activated sludge samples from Sai Kung (SK), Yuen Long (YL), and Tai Po (TP) WWTPs were collected on 5 January 2018. The detailed information on WWTPs and sequenced samples under study are summarized in Table S1 and Table S2, respectively. The operational and physiochemical parameters were provided by the Drainage Services Department of Hong Kong (Table S3).

### Nucleic acid extraction and sequencing

The total DNA of all activated sludge samples was extracted using the FastDNA SPIN Kit for Soil (MP Biomedicals, France), and the total RNA of 12 activated sludge samples from ST WWTPs (April 2017 to March 2018) was extracted using the MoBio RNA PowerSoil total RNA isolation kit (MoBio, USA), following the manufacturer’s instructions. The concentration and quality of the extracted DNA and RNA samples were determined by NanoDrop (Thermo Fisher Scientific, USA) and sequenced on a HiSeq 4000 platform (Illumina, 350 bp insert size, 150 bp paired-end reads). The 13-year ST activated sludge samples yielded a total of 1.0 Tb metagenomic sequencing data. Some of these data had been used to analyze the dynamics of bacteria [16, 21, 22], virome [23], and resistome [18, 24] in our previous studies. In addition to the previously sequenced samples, 23 newly sequenced samples were deposited in the NCBI database under project ID PRJNA432264.

### De novo assembly and binning

Two assembly strategies, namely, single sample assembly and co-assembly (yearly samples), were used in the present study. In brief, metagenomic reads of each sample were assembled independently using metaSPAdes (v3.15.4) [25]. Then, yearly samples were iteratively co-assembled by MEGAHIT (v1.2.9) [26] with default parameters. After the first co-assembly iteration, the clean reads of yearly samples were mapped to the bacterial and archaeal metagenome-assembled genomes (MAGs) retrieved from ST activated sludge samples (see details below) by bowtie2 (v2.4.4) [27]. The mapped prokaryotic reads were discarded from each yearly dataset, and the assembly procedure was repeated with MEGAHIT (v1.2.9) [26]. All assembled contigs (length >1000 bp) were retained for downstream analysis.

The prokaryotic MAGs were integrated from three datasets: (i) 920 MAGs retrieved from the first 9-year samples (2 June 2007 to 3 December 2015, PRJNA432264) [16], (ii) 535 MAGs recovered from samples collected from 5 January 2016 to 5 December 2018, and (iii) 557 MAGs reconstructed from deep-sequenced ST activated sludge samples collected on 5 October 2018 and 1 February 2019 (PRJNA648801) [17]. In the present study, the binning process was performed using “binning” and “refinement” modules of MetaWRAP (v1.2.1) [28]. The total set of 2012 prokaryotic MAGs was dereplicated at an estimated species level using dRep (v3.2.2) [29] with options -pa 0.9, -sa 0.95, -nc 0.30, -cm larger, -comp 50, -con 5. Taxonomy assignment of 1109 dereplicated MAGs was performed using GTDB-Tk (v2.1.1) [30] based on the GTDB database release 214. Likewise, we tried to retrieve eukaryotic MAGs from assembled contigs using the EukRep pipeline. The quality of MAGs was estimated based on marker genes (odb10) using BUSCO (v5.3.2) [31]. The poor genome quality indicated that the reconstruction of eukaryotic genomes from metagenomic data of complex environmental samples remains challenging. Thus, no further eukaryotic genome-based analysis was performed.

### Contig-based analysis

The viral contigs were identified using geNomad (v1.7.4) [32] with default parameters. Sequences shorter than 5000 bp with a virus score lower than 0.9 were left untouched. To identify the metagenomic eukaryotic and prokaryotic contigs in the remaining contigs, two different methods, including Tiara (v1.0.3) [33] and EukRep (v0.6.7) [34], were applied. Only the contig with the same classification by two methods were retained for subsequent analysis. The putative eukaryotic contigs were taxonomically annotated by MetaEuk (v6-a5d39d9) [35] using the NCBI-nr database (downloaded February 2023). We constructed the taxonomic profile of eukaryotes at the superclade and phylum levels. De novo gene prediction was performed using GeneMark-ES (v4.68) [36]. The predicted proteins were clustered with UniRef90 (release 2023_04) [37] MMseqs2/Linclust (v14.7e284) [38] with the options “easy-linclust --min-seq-id 0.9 --cov-mode 1 -c 0.80”. Meanwhile, the predicted proteins were annotated functionally by eggNOG-mapper (v2.1.9) [39] with database 6.0 [40].

Barrnap (v0.9) (https://github.com/tseemann/barrnap) was applied to predict and extract 18S rRNA genes from the assembled scaffolds with the parameter “--kingdom euk”. The eukaryotic V4 region was identified using seqkit amplicon (v2.3.0) [41] using the universal V4 primer set (forward: 5'-CCAGCAGCCGCGGTAATTCC-3'; reverse: 5'-ACTTTCGTTCTTGATTAA-3') The taxonomy of amplicon sequence variants (ASVs) was assigned using QIIME2 pipeline (v2024.5) based on the SILVA SSU database release 138 and visualized using Pavian (v1.0) [42]. Likewise, the rRNA reads were extracted using miTag [43] to estimate the composition of prokaryotes and eukaryotes from metagenomic datasets. The number of extracted rRNA genes was normalized to the average lengths of the 16S (1408 bp) and 18S rRNA (1705 bp) genes in the SILVA SSU database.

### Relative abundances/coverage of eukaryotes, prokaryotes, and viruses in activated sludge samples

The relative abundance/coverage of eukaryotic, prokaryotic, and viral contigs and prokaryotic MAGs was estimated using coverM (v0.6.1) (https://github.com/wwood/CoverM) based on reads mapping (--mapper bwa-mem --min-read-percent-identity 90 --min-read-aligned-percent 80). The multiple-mapping short reads (equally-scored alignments) were determined by minimap2 (v2.23-r1111) and removed by an in-house shell script. Alpha diversity was computed with the diversity function in vegan (v2.6-4) [44]. Non-coding RNA sequences were removed from metatranscriptomic data using SortMeRNA (v4.3.4) [45] based on multiple rRNA databases. The expression of eukaryotes, prokaryotes, and viruses was calculated by mapping the clean metatranscriptomic reads to the corresponding groups using CoverM (v0.6.1) with the aforementioned parameters.

### Ordination analysis and Procrustes analysis

Principal coordinate analysis (PCoA) and non-metric multidimensional scaling (NMDS) analysis were performed on the normalized coverage values (similar to transcripts per million in RNAseq analysis) of the eukaryotic contigs, viral contigs, and prokaryotic MAGs with an occurrence frequency in more than 10% of all samples. NMDS analysis was performed using the metaMDS function in the vegan (v2.6-4) [44] package for R with default parameters. We also tested the congruence among eukaryotic, prokaryotic, and viral populations using Procrustes analysis [46] of NMDS coordinates estimated for each sample with the Bray–Curtis dissimilarity metric (vegan). Procrustes analysis compares the relative positions of points in two multivariate datasets, with the *M*^2^ statistic representing the residual sum of squares after optimal alignment via translation, rotation, and scaling. Significance was tested using the “protest()” function with 999 permutations. Then, coverage values of eukaryotes and prokaryotes were correlated (Pearson correlation) with operation parameters. The result *P* values underwent false discovery correction with the Bonferroni procedure. The varying interaction strength among microbes was inferred using the regularized S-map [20, 47] based on the time series data of both eukaryotic and prokaryotic communities (at the phylum level). Specifically, the microbial interaction strength was represented by the Jacobian element, which was inferred by equation (1). Results were visualized using the ggplot2 package.


(1) \begin{equation*} \hat{c}=\arg \underset{\mathrm{c}}{\min}\frac{1}{N}{\sum}_n^N{w}_n{\left({y}_{n+1}-{X}_nc\right)}^2+\lambda \left(\alpha{\left\Vert c\right\Vert}_2^2+\left(1-\alpha \right){\left|c\right|}_1\right) \end{equation*}

(2) \begin{equation*} {w}_n=\mathit{\exp}\left(\frac{-\theta \left\Vert{X}_n-{X}_n^{\ast}\right\Vert }{\frac{1}{n}{\sum}_n^N\left\Vert{X}_n-{X}_n^{\ast}\right\Vert}\right) \end{equation*}

where $\hat{c}$ is the solution for the weighted least square minimization problem, and *c* is a vector of Jacobian elements. *N* is the total number of time points. The *y*_*n* + 1_ and *X*_n_ are the abundance of a given microbe to be predicted at time point *n* + 1 and observed abundances of all microbes at time point *n*, respectively. The $\lambda \left(\alpha{\left\Vert c\right\Vert}_2^2+\left(1-\alpha \right){\left|c\right|}_1\right)$ is the elastic net penalty [48] and ${w}_n$ is a weight, which is calculated by equation (2). $\left\Vert{X}_n-{X}_n^{\ast}\right\Vert$ is the Euclidian distance between target community state ${X}_n^{\ast }$ at time point *n* and a community state *X_n_* around ${X}_n^{\ast }$ in state space. The $\theta$ is a tuning parameter that determines the region around ${X}_n^{\ast }$ which regression is localized to and is larger than 0. With large $\theta$, only the *X_n_* in close proximity to ${X}_n^{\ast }$ will be used for regression [20].

## Results

### Gene catalog of the eukaryotic microbiome

**Figure 1:**
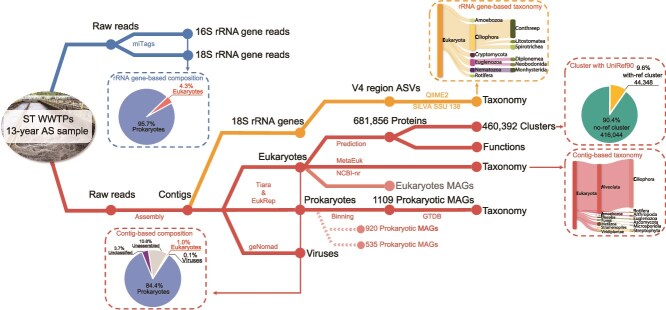
Scheme of the analytical procedure used in this study. The microbial composition was evaluated in terms of rRNA gene-based and contig-based methods, respectively. The eukaryotic taxonomy catalog was determined by 18S rRNA gene V4 region as well as eukaryotic contigs. The eukaryotic gene catalog was established by predicting protein-coding genes from the putative eukaryotic contigs.

From the 143 longitudinal activated sludge samples collected over a span of 13 years, we recovered 1.0 Tb of DNA sequence (Tables S1 and S2). In total, we identified 1 298 761 unique eukaryotic contigs, including 114 807 contigs exceeding 3000 bp in length. Using these contigs, we constructed a eukaryotic protein-coding gene catalog for activated sludge. This catalogue was developed by predicting single- and multi-exons from assembled eukaryotic contigs, resulting in 758 628 protein sequences with lengths over 99 amino acids. Following deduplication, 681 856 non-redundant protein sequences were retained, forming 460 392 protein clusters. The majority of these clusters (90.4%) could not be matched with any of the 176 029 043 clusters from UniRef90 (Fig. 1).

Among the annotated protein-coding genes, we observed that both prokaryotes and eukaryotes encode large amounts of proteins involved in cellular processes and signaling (Fig. S1). Eukaryotic genes, in particular, showed a higher prevalence of protein-coding genes associated with cytoskeleton, RNA processing and modification, and chromatin structure and dynamics, likely due to the complex and dynamic genomic organization in the nucleus in eukaryotes. Additionally, among the annotated eukaryotic protein-coding genes, nearly 2000 genes (0.5%) were found to encode enzymes involved in cellulose degradation, such as cellulase, cellulose binding, cellobiosidase, glucanase, and glucosidase. This suggests eukaryotes could contribute to the removal of cellulose-rich organic matter that tends to resist bacterial decay. In contrast, enzymes related to metabolism — such as those involved in energy production and conversion, biosynthesis of amino acids, coenzymes, lipids, as well as inorganic ion transport and metabolisms—were enriched in prokaryotes (Fig. S1). As expected, they are the primary populations in activated sludge directly associated with fundamental functions of WWTPs, such as organic matter and nutrient removal.

### Populations in activated sludge samples

Eukaryotic contigs accounted for 1.0% (± 0.4%) of all clean reads (Fig. 1), a portion significantly lower than that observed in the rRNA gene-based analysis (4.3 ± 2.4%, *P* value <0.001, Fig. 1). Of the putative eukaryotic contigs, 19.7% (256 121) could be taxonomically assigned, representing 3 kingdoms (Metazoa, Viridiplantae, and Fungi) across 49 phyla. The Stramenopila–Alveolata–Rhizaria (SAR) group, with 148 484 contigs, dominated the annotated eukaryotic contigs, with 96.1% of these classified into the phylum of Ciliophora (Fig. 1), primarily comprising Spirotrichea, Oligohymenophorea, and Heterotrichea. This finding aligns with the known prevalence of protozoa in activated sludge, where ~70% are ciliates [49]. These are the most common predators of bacteria and can also feed on suspended particles, contributing to higher effluent quality.

Among the other abundant eukaryotic groups, Metazoa was well represented with 34 961 contigs assigned to phyla such as Rotifera (12 191, rotifers), Chordata (4541, chordates), Arthropoda (3684, arthropods), and Nematoda (3486, nematodes). The kingdom of Viridiplantea (9836, plants) was almost exclusively represented by Streptophyta (9228, algae and plants), whereas the kingdom Fungi (5232) was primarily composed of Ascomycota (1252) and Microsporidia (1108). In 18S rRNA gene-based analysis, we only identified 397 eukaryotic ASVs from 143 activated sludge samples based on V4 regions, and their taxonomic distribution was in agreement with the contig-based result (Table S4 and Fig. S2).

In addition to eukaryotic contigs, we also retrieved 44 467 353 prokaryotic contigs and 109 913 viral contigs from all activated sludge samples, representing 84.4% (± 3.4%) and 0.1% (± 0.0%) of the total reads, respectively (Fig. 1). This distribution reflects the known dominance of prokaryotes in activated sludge samples, being mainly responsible for wastewater cleanup. We reconstructed 1109 dereplicated prokaryotic MAGs (Table S5 and Fig. S3), spanning 46 phyla, which were dominated by *Pseudomonadota* (305 MAGs), *Bacteroidota* (166), *Patescibacteria* (118), and *Planctomycetota* (98). Additionally, viral contigs were dominated by bacteriophages, and 97.1% were assigned to the class of *Caudoviricetes*. Likewise, 10.8% of the DNA reads remained unassembled, primarily consisting of rare microbes that could not be effectively assembled. Moreover, 3.7% of total reads were equally mapped to eukaryotes, prokaryotes, or viruses, and were therefore denoted as unclassified.

### Temporal dynamics of microbial populations in activated sludge samples

**Figure 2:**
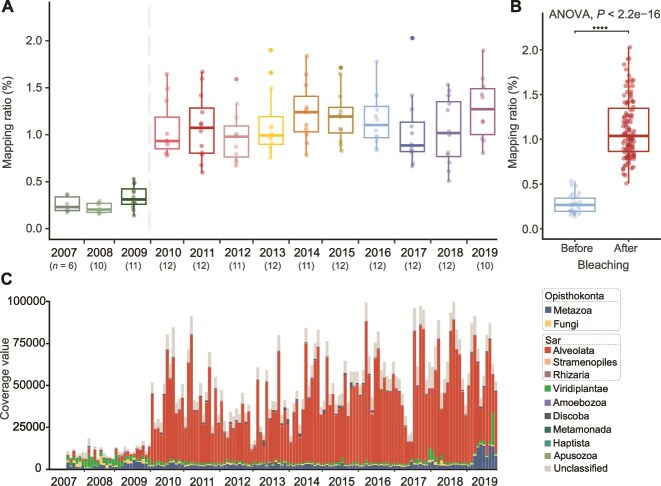
Temporal dynamics of eukaryotic populations in activated sludge samples for 13 years (A). The mapping ratio was calculated based on the ratio of recruited reads of the eukaryotic contigs. The “*n*” corresponds to the sample number of each year available for analysis. Relative abundance of eukaryotic populations before and after the bleaching event. Significance was determined with a multivariate non-parametric ANOVA (B). Eukaryotic taxonomy dynamic of activated sludge over 13 years (C). The coverage value was normalized by contig length and library size, which is similar to transcripts per million (TPM) in RNAseq analysis.

The dynamics of the activated sludge community exhibit a distinctive feature that the relative abundance of eukaryotic populations quadrupled after the primary bleaching event in December 2009 (*P* value <0.001), where the studied WWTPs experienced seasonal foaming (Figs 2A, B and S4). Following this event, the eukaryotes fluctuated around a stable average of 1.1 ± 0.3% for 10 years. The sharp increase was primarily driven by Ciliophora, which showed a 27-fold difference in relative abundance before and after the transient intervention (Fig. 2C). In addition, at the phylum level, the relative abundance of Arthropoda, Euglenozoa, Mucoromycota, Evosea, Cnidaria, and Bacillariophyta significantly increased (*P* value <0.001), while Streptophyta, Rotifera, and Microsporidia declined (Fig. S5).

**Figure 3:**
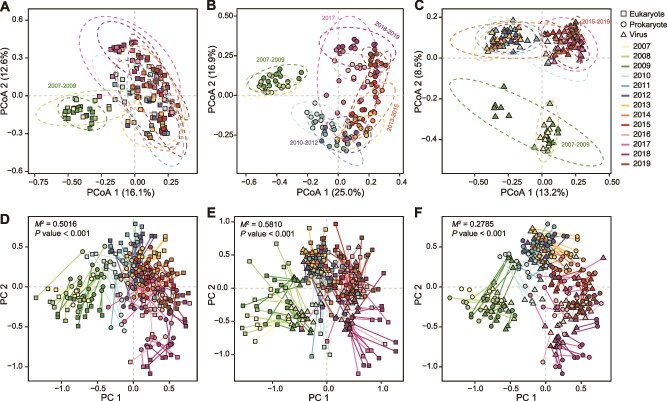
Principal coordinate analysis (PCoA) of activated sludge samples based on Bray–Curtis distances of eukaryotes (A), prokaryotes (B), and viruses (C). Ellipsoids represent a 95% confidence interval surrounding each group. The congruences between eukaryotes and prokaryotes (D), eukaryotes and viruses (E), and prokaryotes and viruses (F) were depicted by the Procrustes analysis. Each metagenome was represented by one pair of nodes in a square (eukaryote), a circle (prokaryote), and a triangle (virus). *M*^2^ and *P* values were shown to indicate the robustness of the correlation.

The bleaching intervention efficiently alleviated the biological foaming and also shifted the composition of the prokaryotic community from a predominance of *Actinomycetota* to *Pseudomonadota*, as we demonstrated in a previous study [16]. This is also in agreement with previous findings that the addition of sodium hypochlorite could introduce a significant change in microbial communities [50, 51]. Additionally, the sewage under study is saline wastewater with a salinity of 1.2% due to seawater toilet flushing. However, there was a sudden decrease in wastewater salinity in August 2017, which persisted for 11 months. Associated with this disturbance, a population transition across eukaryotes, prokaryotes, and viruses (Fig. 3A-C) at lower taxonomic levels was observed although higher taxonomic levels remained stable (Figs 2 and S6). For example, a prokaryotic species turnover occurred along salinity disturbance, whereas higher taxonomic levels appeared unchanged (Fig. S6). Unlike prokaryotic populations [16, 52], however, no successional dynamics were observed in the relative abundance of eukaryotic populations on monthly or seasonal timescales (Fig. S7).

PCoA analysis revealed temporally similarly structured clusters of eukaryotes, prokaryotes, and viruses (Fig. 3A-C), prompting us to investigate the relationship among them. Significant correlations among eukaryotic, prokaryotic, and viral populations were observed based on Procrustes analysis (*P* value <0.001, Fig. 3D-F), with particularly strong associations between prokaryotes and viruses (*M*^2^ = 0.28). Because viral contigs are dominated by bacteriophages, this finding was consistent with the phage-host dynamics observed in a hybrid moving bed biofilm reactor [53]. In contrast, eukaryotes and viruses showed a weaker correlation, likely because the eukaryotic virome is dominated by RNA viruses [54].

Overall, our findings could indicate that external factors, such as transient intervention (bleaching) or gradual changes (mean cell residence time and salinity), are closely associated with community shifts. In spite of these changes that showed covariation among eukaryotes, prokaryotes, and viruses, the pollutant removal rates remained relatively stable over the 13-year observation period (e.g. COD: 87.6 ± 3.8%; NH_4_^+^-N: 92.6 ± 6.8%, Table S3). This suggests that even though the taxonomic composition and environmental conditions changed, the microbial community in activated sludge could maintain functional stability (Figs 3, S6, and S11).

The diversity of eukaryotes temporarily decreased during the period of lower salinity, disturbing a trend of increasing diversity seen in the richness (Fig. S8). In contrast, the richness and diversity of prokaryotes remained relatively stable over 11 years (2007–2017), although the salinity disturbance seems to bring a decline in diversity. In addition, considering the successional dynamics on long-time scales (Fig. 3), species coexistence and high biodiversity allow plasticity in response to changing environments. Moreover, as expected, Pielou’s evenness revealed a seasonal trend with the highest values identified in summer (Fig. S9), implying that warmer temperatures appeared to contribute to higher biodiversity for eukaryotes, which is consistent with the seasonal patterns found for bacterial studies [16, 52].

### Environmental drivers and interactions of activated sludge microbiome

**Figure 4:**
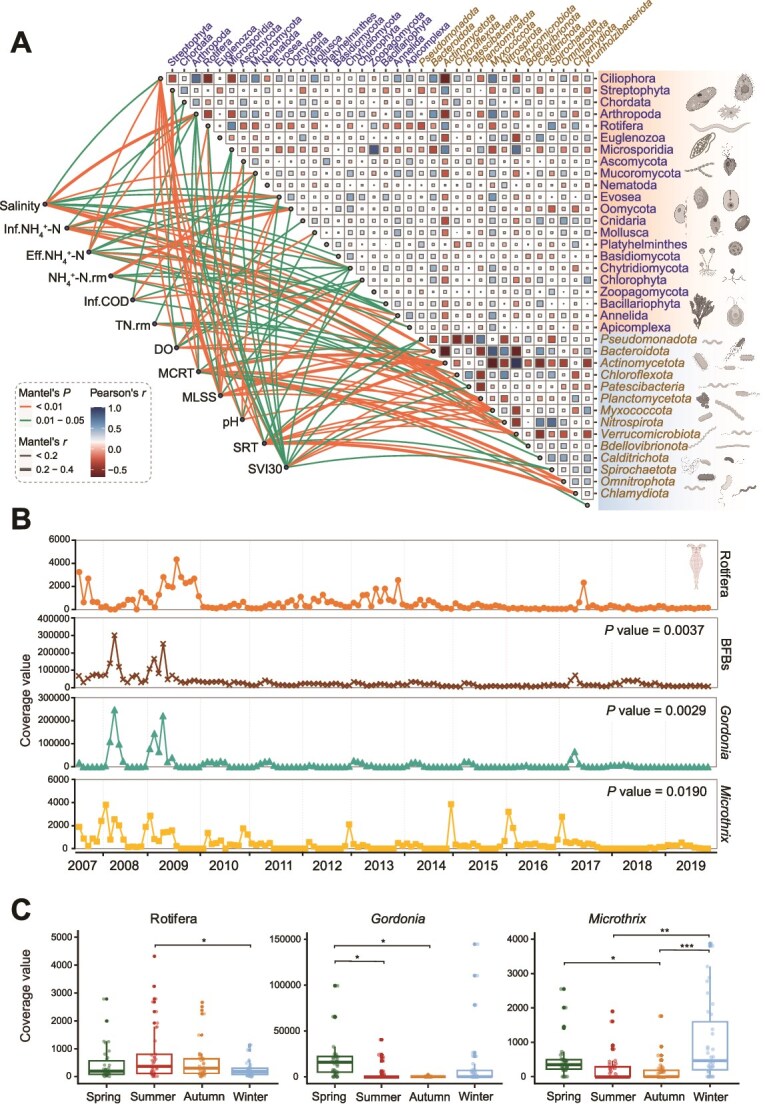
Relationships between microorganisms and environmental variables (A). Edge width and color correspond to Mantel’s *r* value and statistical significance, respectively. Pairwise correlations of microorganisms are shown with a color gradient denoting Pearson’s correlation coefficient. Inf.: influent; Eff.: effluent; NH_4_^+^-N: ammonia nitrogen; TN: total nitrogen; COD: chemical oxygen demand; rm: removal rate; DO: dissolved oxygen; MCRT: mean cell residence time; MLSS: mixed liquor suspended solids; SRT: sludge retention time; SVI30: sludge volume index at 30 min. Temporal (B) and seasonal (C) dynamics of Rotifera and bulking and foaming (BFB) bacteria over 13 years. The cross-correlation analysis was performed between Rotifera and each bulking and foaming bacterial species; only the species with significant correlation and the seasonal pattern was shown in the figure.

NMDS ordination analysis was used to find the operation parameters and environmental variables that best explained the patterns of community dynamics over time (Figs S10 and S11). This indicated that the distribution of the eukaryotes and prokaryotes was primarily associated with salinity, influent chemical oxygen demand (COD) and total nitrogen (TN) concentrations, and ammonia removal (Bonferroni-corrected *P* value <0.05). Furthermore, environmental variables, such as temperature, rainfall, and humidity, played a limited role in driving microbial dynamics. In contrast, microbial community structure showed strong correlations with sludge retention time, mean cell residence time (MCRT), and mixed liquor suspended solids (Fig. 4A), which are closely related, illustrating the importance of these parameters in shaping the communities, maintaining the functions, and managing WWTPs [55, 56].

Growing evidence has indicated that microbial interaction in complex communities follows a varying and nonlinear pattern [47, 57]. Therefore, we also applied an empirical dynamic modeling approach (i.e. regularized S-map) to investigate the microbial interactions, which generated the relevant Jacobian elements that defined the interaction strengths. Based on the large positive interaction strengths, bacteria appear to enhance their reproduction to promote overall community growth (Fig. S12). The relatively weak positive interactions among *Patescibacteria* can be partially explained by the characteristics of the ultra-small *Patescibacteria*, which are adapted to oligotrophic environments with minimal genomes and low growth rates [58, 59]. Comparably, weak interaction strengths were observed within eukaryotic phyla, probably owing to their physiological complexity and low growth rate. Additionally, the two most abundant bacterial phyla (Fig. 4a), namely, *Pseudomonadota* (relative abundance: 25.7 ± 4.9%) and *Bacteroidota* (10.0 ± 3.9%), displayed positive correlations with most eukaryotes, in contrast to the behavior observed in *Actinomycetota*. In addition, Streptophyta, Rotifer, and Microsporidia exhibited a significant negative correlation with most eukaryotic phyla, and the relative abundance of species in those phyla sharply decreased after the bleaching intervention whereas other eukaryotes bloomed. Likewise, asymmetric interactions were found between Arthropoda and Euglenozoa in terms of Jacobian elements, implying a predator role of Arthropoda (Fig. S12). In addition, similar exploitation or predator–prey relationships were also inferred between, Ascomycota and *Patescibacteria*, Mucoromycota and *Bdellovobrionota*, as well as Rotifera and *Actinomycetota*. Even though the majority of fungi have saprophytic or symbiotic lifestyles, a small portion of Ascomycota and Mucoromycota represent carnivorous fungi that possess the ability to trap and digest prey [60]. At the lower taxonomic levels (eukaryotic phylum and bacterial species), Rotifera exhibited significant positive correlations (*P* value <0.01) and opposite seasonal pattern with bulking and foaming bacterial species affiliated with the genera of *Gordonia* and *Microthrix* based on cross-correlation analysis (Fig. 4B and C). *Gordonia* and *Microthrix* revealed seasonal fluctuations with higher abundances reported in winter and spring, in agreement with our previous studies [61, 62], while Rotifera flourished in summer and autumn. Our findings provided a high resolution of cross-domain interactions in activated sludge. Leveraging the knowledge of microbial ecology could enable strategies to engineer wastewater microbes and contribute to operational issues.

### Expression of eukaryotic populations in Hong Kong WWTPs

In the metatranscriptomic dataset, 6.3% (± 3.8%) of rRNA-derived and non-coding RNA sequences were filtered out (Fig. S13). Eukaryotic transcriptomes accounted for 2.0% ± (0.7%) of total filtered RNA reads, a portion significantly higher than that of eukaryotic DNA reads (1.0 ± 0.4%) and consistent with previous studies [63, 64]. In contrast, prokaryotes were the most abundant population that displayed relatively low transcriptional expression (Fig. S13). Because eukaryotes divide less frequently, their mRNA exhibits long half-lives ranging from several minutes to more than a day [65, 66], and, in comparison, fast mRNA degradation of prokaryotes can favor them to respond to changing environments promptly [64, 67]. Of the taxonomy-assigned eukaryotic contigs, Ciliophora and Arthropoda had low transcriptional expressions (RNA/DNA <1) over the year, whereas opposite trends were observed in Streptophyta and Mucoromycota (Fig. S14, *P* value <0.01). The RNA/DNA ratio of Rotifera also displayed an apparent seasonal pattern with high transcriptional levels identified in summer, supporting the result of metagenomic analysis. Likewise, Euglenozoa, Chlorophyta, Apicomplexa, and Evosea showed high transcriptional activities in spring and summer, likely related to their trophic types, increased temperature, and MCRT (Table S3). For example, the majority of Euglenozoa and Chlorophyta in activated sludge are phototrophs possessing chloroplast(s) [68, 69], which favors their growth in warm seasons. Apicomplexa primarily comprises parasitic alveolates [70], whose high transcriptional expressions are also associated with hosts’ activities. These observations provided transcriptional evidence and novel insights into eukaryotic activities in the activated sludge ecosystem.

### Biogeographic dynamics of eukaryotic populations in Hong Kong WWTPs

**Figure 5:**
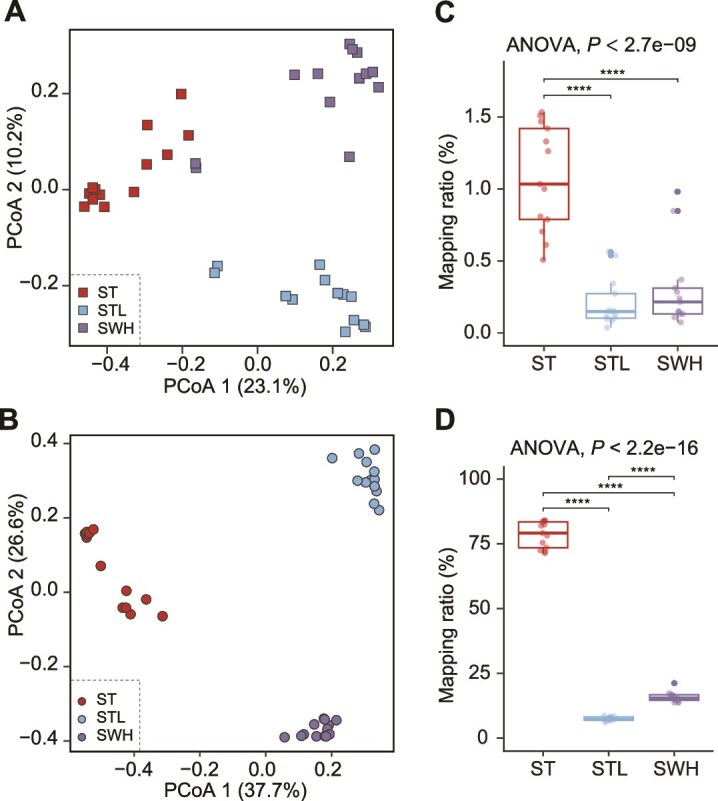
Biogeographic diversity of three local WWTPs over one-year monitoring based on the Bray–Curtis distances of eukaryotes (A) and prokaryotes (B). Each dot represented the microbial community structure of an individual sample. Comparisons of eukaryotes (C) and prokaryotes (D) ratios in three local WWTPs (Bonferroni-corrected *P* value <0.05).

In addition to Sha Tin (ST) WWTPs, five additional secondary WWTPs, including Stanley (STL), Tai Po (TP), Yuen Long (YL), Shek Wu Hui (SWH), and Sai Kung (SK) WWTPs, were selected to analyze the biogeographic distributions of eukaryotes in activated sludge ecosystems (Fig. S15). Generally, the microbial population was distinct at each site, especially for STL and TP WWTPs. Among six WWTPs, both ST and TP WWTPs treated saline wastewater (Table S1). In Hong Kong, seawater toilet flushing is used by ~80% of the residents to relieve water scarcity, and the wastewater in ST WWTPs comprised ~30% of seawater, resulting in a sewage salinity of ~1.2%. In comparison, the other four WWTPs treated fresh municipal wastewater, whereas both eukaryotic and prokaryotic communities in YL were affected by industrial wastewater (Fig. S15). In a one-year monitoring period (Fig. 5), the prokaryotes in ST, STL, and SWH were significantly different from each other, probably owing to the wastewater salinity as well as different treatment technologies. Namely, STL adopts a hybrid of activated sludge and moving bed biofilm. Meanwhile, saline WWTPs represent a special ecosystem comprising a unique eukaryotic population, which is distinct from that of fresh WWTPs. Taken together, the results indicated that eukaryotic and prokaryotic populations were geographically diverse in large part primarily associated with the sewage composition, such as seawater and industrial wastewater, as well as treatment technologies.

## Discussion

Eukaryotic populations are usually overlooked in metagenomic studies largely due to insufficient sequencing depth. Here, we performed a metagenomics analysis of >1.0 Tb sequencing data and constructed a comprehensive eukaryotic taxonomy and gene catalog for activated sludge. More than 90% of protein clusters and ~ 80% of eukaryotic contigs remained unannotated or could not be taxonomically assigned using existing databases. This highlights the vast, uncharted functional potential of eukaryotic microbiomes in activated sludge ecosystems. Our longitudinal dynamics of the three important microbial components (prokaryotes, eukaryotes, and viruses) of the activated sludge enabled a describe ~90% of microbes in this complex ecosystem. A synchronous succession of eukaryotes, prokaryotes, and viruses was revealed through Procrustes analysis in this dynamic environment. The species in these groups were closely linked and co-varied possibly via co-occupancy of habitats and interactions in food chains, such as predation, cooperation, and competition. Additionally, only DNA viruses (phages) were sequenced in the present study, leading to a lower correlation among eukaryotes and viruses compared to prokaryotes and viruses. Cross-domain interactions among eukaryotes and prokaryotes were revealed through our metagenomic analysis. At the phylum level, stronger positive interaction strengths were observed among prokaryotes compared with eukaryotes. Furthermore, the predator–prey/exploitation relationship among eukaryotes and prokaryotes was elucidated through regularized S-map and cross-correlation analysis, illustrating the predatory role of Arthropoda, Rotifera, Ascomycota, and Mucoromycota in activated sludge. These findings indicated that eukaryotes, usually with large body sizes and complex structures and occupy higher trophic levels, are ecologically important microbes in activated sludge even though they were rare populations.

The operational parameters significantly influenced the taxonomy composition and relative abundance of both eukaryotes and prokaryotes, especially in response to transient intervention (bleaching) and intermittent fluctuations (MCRT and salinity). Specifically, bleach solution was added to the studied WWTPs in December 2009 to mitigate the seasonal foaming, resulting in a pronounced shift among both eukaryotes and prokaryotes. The dominant prokaryotic phylum shifted from *Actinomycetota* to *Pseudomonadota*, consistent with our previous reports [16, 52], while the Ciliophora became the most abundant eukaryotic phylum after the bleaching event. Even though it is widely understood that bleaching treatment substantially alters bacterial community composition [50, 51], the mechanism of eukaryotes in response to the transient bleaching intervention remains largely unclear. Likewise, salinity fluctuations were associated with declining diversity in both eukaryotes and prokaryotes and introduced a lower-level taxonomic turnover whereas the higher taxonomic levels remained stable at the phylum level for prokaryotes and the kingdom/clade level for eukaryotes. Finally, the biogeographic distributions of eukaryotes and prokaryotes suggested that salinity, industrial wastewater, and treatment technology could also contribute to microbial community composition in municipal WWTPs. However, the associations between longitudinal and biogeographic eukaryotic dynamics and operational parameters could not explain the underlying mechanisms and require further rigorous lab-scale studies to provide unequivocal evidence.

The rarefaction analysis indicates that despite the extensive sampling effort and sequencing depth, eukaryote species diversity has not approached saturation, underscoring the need for further research (Fig. S16). We expected that targeted sequencing could address this issue soon. Moreover, the relative abundance of eukaryotes reported in this study was much lower than the estimated abundance reported in previous studies [8, 9], probably owing to the inherent limitations of different methods. For instance, a large range of cell wall structures of eukaryotes could result in inefficient DNA extraction and underestimation, whereas biomass-based estimation using microscopy could be affected by biases introduced by the observers, sampling, or conformation. Additionally, the removal of multiple-mapped reads when determining the abundances likely contributed to a considerable underestimation of rare microbes, especially for eukaryotes and viruses. For example, our result showed that more than 60% of mapped “eukaryotic reads” could be equally aligned to prokaryotes, and this ratio could reach an even higher level of 95% for viruses and prokaryotes. Likewise, those multiple-mapping reads would result in significant but misleading positive correlations among different communities. Therefore, these multiple-mapping reads, probably encoding universal proteins, were worthy of careful consideration during the abundance determination in metagenomic studies. With the advancement of long-read sequencing technologies, we evaluated the microbial composition in two nanopore-sequenced ST activated sludge samples. As expected, the ratio of unclassified reads decreased from 3.7% to 0.2%, which demonstrates that the multiple-mapping issue is expected to be improved in the near future.

## Supplementary Material

EUK_MS_SI_V10_wraf058

supplementary_V10_wraf058

## Data Availability

The raw nucleotide sequence data used in the present study were collected from the NCBI database of project ID PRJNA432264. The newly sequenced raw nucleotide sequence data have been deposited in the NCBI database under the same project ID. Details are included within the article and supplementary files.

## References

[1] Lawson CE, Harcombe WR, Hatzenpichler R. et al. Common principles and best practices for engineering microbiomes. *Nat Rev Microbiol* 2019;17:725–41. 10.1038/s41579-019-0255-9 31548653 PMC8323346

[2] Saunders AM, Albertsen M, Vollertsen J. et al. The activated sludge ecosystem contains a core community of abundant organisms. *ISME J* 2016;10:11–20. 10.1038/ismej.2015.117 26262816 PMC4681854

[3] Seviour R, Nielsen PH. Microbial Ecology of Activated Sludge: IWA Publishing. London: IWA Publishing, 2010, 10.2166/9781780401645

[4] Matsunaga K, Kubota K, Harada H. Molecular diversity of eukaryotes in municipal wastewater treatment processes as revealed by 18S rRNA gene analysis. *Microbes Environ* 2014;29:401–7. 10.1264/jsme2.ME14112 25491751 PMC4262364

[5] Madoni P . Protozoa in wastewater treatment processes: a minireview. *Ital J Zool* 2011;78:3–11. 10.1080/11250000903373797

[6] Foissner W . Protists as bioindicators in activated sludge: identification, ecology and future needs. *Eur J Protistol* 2016;55:75–94. 10.1016/j.ejop.2016.02.004 27062305

[7] Gray NF . Activated Sludge: Developments and Sustainable Solutions. Singapore: World Scientific, 2023. 10.1142/q0408.

[8] Curds CR, Hawkes HA. Ecological Aspects of Used-Water Treatment, Vol. I. London: Academic Press, 1975.

[9] Pike E, Curds C. The Microbial Ecology of the Activated Sludge Process. New York: Academic Press, 1971. 10.1016/B978-0-12-648050-4.50012-2 4347114

[10] Curds CR, Cockburn A. Protozoa in biological sewage-treatment processes—II. Protozoa as indicators in the activated-sludge process. *Water Res* 1970;4:237–49. 10.1016/0043-1354(70)90070-9

[11] Salvado H, Gracia M, Amigó J. Capability of ciliated protozoa as indicators of effluent quality in activated sludge plants. *Water Res* 1995;29:1041–50. 10.1016/0043-1354(94)00258-9

[12] Perez-Uz B, Arregui L, Calvo P. et al. Assessment of plausible bioindicators for plant performance in advanced wastewater treatment systems. *Water Res* 2010;44:5059–69. 10.1016/j.watres.2010.07.024 20678787

[13] Cohen Y, Pasternak Z, Johnke J. et al. Bacteria and microeukaryotes are differentially segregated in sympatric wastewater microhabitats. *Environ Microbiol* 2019;21:1757–70. 10.1111/1462-2920.14548 30702191

[14] Herrera ML, Vallor AC, Gelfond JA. et al. Strain-dependent variation in 18S ribosomal DNA copy numbers in *aspergillus fumigatus*. *J Clin Microbiol* 2009;47:1325–32. 10.1128/jcm.02073-08 19261786 PMC2681831

[15] Prokopowich CD, Gregory TR, Crease TJ. The correlation between rDNA copy number and genome size in eukaryotes. *Genome* 2003;46:48–50. 10.1139/g02-103 12669795

[16] Wang Y, Ye J, Ju F. et al. Successional dynamics and alternative stable states in a saline activated sludge microbial community over 9 years. *Microbiome.* 2021;9:199. 10.1186/s40168-021-01151-5 34615557 PMC8495973

[17] Liu L, Wang Y, Yang Y. et al. Charting the complexity of the activated sludge microbiome through a hybrid sequencing strategy. *Microbiome* 2021;9:205. 10.1186/s40168-021-01155-1 34649602 PMC8518188

[18] Yin X, Yang Y, Deng Y. et al. An assessment of resistome and mobilome in wastewater treatment plants through temporal and spatial metagenomic analysis. *Water Res* 2022;209:117885. 10.1016/j.watres.2021.117885 34847392

[19] Cenci S, Sugihara G, Saavedra S. Regularized S-map for inference and forecasting with noisy ecological time series. *Methods Ecol Evol* 2019;10:650–60. 10.1111/2041-210X.13150

[20] Deyle ER, May RM, Munch SB. et al. Tracking and forecasting ecosystem interactions in real time. *Proc R Soc B Biol Sci* 2016;283:20152258. 10.1098/rspb.2015.2258 PMC4721089 26763700

[21] Wang Y, Qin W, Jiang X. et al. Seasonal prevalence of ammonia-oxidizing archaea in a full-scale municipal wastewater treatment plant treating saline wastewater revealed by a 6-year time-series analysis. *Environ Sci Technol* 2021;55:2662–73. 10.1021/acs.est.0c07703 33539079

[22] Wang Y, Zhang Y, Hu Y. et al. Genome-centric metagenomics reveals the host-driven dynamics and ecological role of CPR bacteria in an activated sludge system. *Microbiome* 2023;11:56. 10.1186/s40168-023-01494-1 36945052 PMC10031880

[23] Wang Y, Jiang X, Liu L. et al. High-resolution temporal and spatial patterns of virome in wastewater treatment systems. *Environ Sci Technol* 2018;52:10337–46. 10.1021/acs.est.8b03446 30148618

[24] Yin X, Deng Y, Ma L. et al. Exploration of the antibiotic resistome in a wastewater treatment plant by a nine-year longitudinal metagenomic study. *Environ Int* 2019;133:105270. 10.1016/j.envint.2019.105270 31683155

[25] Nurk S, Meleshko D, Korobeynikov A. et al. metaSPAdes: a new versatile metagenomic assembler. *Genome Res* 2017;27:824–34. 10.1101/gr.213959.116 28298430 PMC5411777

[26] Li D, Liu C-M, Luo R. et al. MEGAHIT: an ultra-fast single-node solution for large and complex metagenomics assembly via succinct de Bruijn graph. *Bioinformatics* 2015;31:1674–6. 10.1093/bioinformatics/btv033 25609793

[27] Langmead B, Salzberg SL. Fast gapped-read alignment with bowtie 2. *Nat Methods* 2012;9:357–9. 10.1038/nmeth.1923 22388286 PMC3322381

[28] Uritskiy GV, DiRuggiero J, Taylor J. MetaWRAP—a flexible pipeline for genome-resolved metagenomic data analysis. *Microbiome.* 2018;6:158. 10.1186/s40168-018-0541-1 30219103 PMC6138922

[29] Olm MR, Brown CT, Brooks B. et al. dRep: a tool for fast and accurate genomic comparisons that enables improved genome recovery from metagenomes through de-replication. *ISME J* 2017;11:2864–8. 10.1038/ismej.2017.126 28742071 PMC5702732

[30] Chaumeil P-A, Mussig AJ, Hugenholtz P. et al. GTDB-Tk v2: memory friendly classification with the genome taxonomy database. *Bioinformatics* 2022;38:5315–6. 10.1093/bioinformatics/btac672 36218463 PMC9710552

[31] Simão FA, Waterhouse RM, Ioannidis P. et al. BUSCO: assessing genome assembly and annotation completeness with single-copy orthologs. *Bioinformatics* 2015;31:3210–2. 10.1093/bioinformatics/btv351 26059717

[32] Camargo AP, Roux S, Schulz F. et al. Identification of mobile genetic elements with geNomad. *Nat Biotechnol* 2024;42:1303–12. 10.1038/s41587-023-01953-y 37735266 PMC11324519

[33] Karlicki M, Antonowicz S, Karnkowska A. Tiara: deep learning-based classification system for eukaryotic sequences. *Bioinformatics* 2022;38:344–50. 10.1093/bioinformatics/btab672 34570171 PMC8722755

[34] West PT, Probst AJ, Grigoriev IV. et al. Genome-reconstruction for eukaryotes from complex natural microbial communities. *Genome Res* 2018;28:569–80. 10.1101/gr.228429.117 29496730 PMC5880246

[35] Levy Karin E, Mirdita M, Söding J. MetaEuk—sensitive, high-throughput gene discovery, and annotation for large-scale eukaryotic metagenomics. *Microbiome.* 2020;8:48. 10.1186/s40168-020-00808-x 32245390 PMC7126354

[36] Lomsadze A, Ter-Hovhannisyan V, Chernoff YO. et al. Gene identification in novel eukaryotic genomes by self-training algorithm. *Nucleic Acids Res* 2005;33:6494–506. 10.1093/nar/gki937 16314312 PMC1298918

[37] Suzek BE, Huang H, McGarvey P. et al. UniRef: comprehensive and non-redundant UniProt reference clusters. *Bioinformatics* 2007;23:1282–8. 10.1093/bioinformatics/btm098 17379688

[38] Steinegger M, Söding J. MMseqs2 enables sensitive protein sequence searching for the analysis of massive data sets. *Nat Biotechnol* 2017;35:1026–8. 10.1038/nbt.3988 29035372

[39] Cantalapiedra CP, Hernández-Plaza A, Letunic I. et al. eggNOG-mapper v2: functional annotation, orthology assignments, and domain prediction at the metagenomic scale. *Mol Biol Evol* 2021;38:5825–9. 10.1093/molbev/msab293 34597405 PMC8662613

[40] Hernández-Plaza A, Szklarczyk D, Botas J. et al. eggNOG 6.0: enabling comparative genomics across 12 535 organisms. *Nucleic Acids Res* 2023;51:D389–94. 10.1093/nar/gkac1022 36399505 PMC9825578

[41] Shen W, Le S, Li Y. et al. SeqKit: a cross-platform and ultrafast toolkit for FASTA/Q file manipulation. *PLoS One* 2016;11: e0163962. 10.1371/journal.pone.0163962 27706213 PMC5051824

[42] Breitwieser FP, Salzberg SL. Pavian: interactive analysis of metagenomics data for microbiome studies and pathogen identification. *Bioinformatics* 2020;36:1303–4. 10.1093/bioinformatics/btz715 31553437 PMC8215911

[43] Logares R, Sunagawa S, Salazar G. et al. Metagenomic 16S rDNA Illumina tags are a powerful alternative to amplicon sequencing to explore diversity and structure of microbial communities. *Environ Microbiol* 2014;16:2659–71. 10.1111/1462-2920.12250 24102695

[44] Oksanen J, Kindt R, Legendre P. et al. The vegan package. *Community ecology package* 2007;10:719.

[45] Kopylova E, Noé L, Touzet H. SortMeRNA: fast and accurate filtering of ribosomal RNAs in metatranscriptomic data. *Bioinformatics* 2012;28:3211–7. 10.1093/bioinformatics/bts611 23071270

[46] Gower JC . Generalized procrustes analysis. *Psychometrika* 1975;40:33–51. 10.1007/BF02291478

[47] Yu Z, Gan Z, Huang H. et al. Regularized S-map reveals varying bacterial interactions. *Appl Environ Microbiol* 2020;86:e01615–20. 10.1128/AEM.01615-20 32801185 PMC7531961

[48] Zou H, Hastie T. Regularization and variable selection via the elastic net. *Journal of the Royal Statistical Society Series B: Statistical Methodology* 2005;67:301–20. 10.1111/j.1467-9868.2005.00503.x

[49] Ratsak C, Maarsen K, Kooijman S. Effects of protozoa on carbon mineralization in activated sludge. *Water Res* 1996;30:1–12. 10.1016/0043-1354(95)00096-4

[50] Navarro RR, Hori T, Inaba T. et al. High-resolution phylogenetic analysis of residual bacterial species of fouled membranes after NaOCl cleaning. *Water Res* 2016;94:166–75. 10.1016/j.watres.2016.02.044 26945453

[51] Han X, Wang Z, Wang X. et al. Microbial responses to membrane cleaning using sodium hypochlorite in membrane bioreactors: cell integrity, key enzymes and intracellular reactive oxygen species. *Water Res* 2016;88:293–300. 10.1016/j.watres.2015.10.033 26512807

[52] Ju F, Guo F, Ye L. et al. Metagenomic analysis on seasonal microbial variations of activated sludge from a full-scale wastewater treatment plant over 4 years. *Env Microbiol Rep* 2014;6:80–9. 10.1111/1758-2229.12110 24596265

[53] Wang D, Liu L, Xu X. et al. Distributions, interactions, and dynamics of prokaryotes and phages in a hybrid biological wastewater treatment system. *Microbiome.* 2024;12:134. 10.1186/s40168-024-01853-6 39039555 PMC11265110

[54] Koonin EV, Dolja VV, Krupovic M. Origins and evolution of viruses of eukaryotes: the ultimate modularity. *Virology* 2015;479-480:2–25. 10.1016/j.virol.2015.02.039 25771806 PMC5898234

[55] Akarsubasi AT, Eyice O, Miskin I. et al. Effect of sludge age on the bacterial diversity of bench scale sequencing batch reactors. *Environ Sci Technol* 2009;43:2950–6. 10.1021/es8026488 19475976

[56] Jiang X, Ye L, Ju F. et al. Temporal dynamics of activated sludge bacterial communities in two diversity variant full-scale sewage treatment plants. *Appl Microbiol Biotechnol* 2018;102:9379–88. 10.1007/s00253-018-9287-8 30099572

[57] Zhao Y, Liu Z, Zhang B. et al. Inter-bacterial mutualism promoted by public goods in a system characterized by deterministic temperature variation. *Nat Commun* 2023;14:5394. 10.1038/s41467-023-41224-7 37669961 PMC10480208

[58] Tian R, Ning D, He Z. et al. Small and mighty: adaptation of superphylum *Patescibacteria* to groundwater environment drives their genome simplicity. *Microbiome.* 2020;8:51. 10.1186/s40168-020-00825-w 32252814 PMC7137472

[59] Chaudhari NM, Overholt WA, Figueroa-Gonzalez PA. et al. The economical lifestyle of CPR bacteria in groundwater allows little preference for environmental drivers. *Environ microbiome* 2021;16:24. 10.1186/s40793-021-00395-w 34906246 PMC8672522

[60] Yang E, Xu L, Yang Y. et al. Origin and evolution of carnivorism in the Ascomycota (fungi). *Proc Natl Acad Sci USA* 2012;109:10960–5. 10.1073/pnas.1120915109 22715289 PMC3390824

[61] Jiang X, Guo F, Zhang T. Population dynamics of bulking and foaming bacteria in a full-scale wastewater treatment plant over five years. *Sci Rep* 2016;6:24180. 10.1038/srep24180 27064107 PMC4827064

[62] Jiang X, Ye L, Ju F. et al. Toward an intensive longitudinal understanding of activated sludge bacterial assembly and dynamics. *Environ Sci Technol* 2018;52:8224–32. 10.1021/acs.est.7b05579 29943968

[63] Freudenthal J, Ju F, Bürgmann H. et al. Microeukaryotic gut parasites in wastewater treatment plants: diversity, activity, and removal. *Microbiome.* 2022;10:27. 10.1186/s40168-022-01225-y 35139924 PMC8827150

[64] Yu K, Zhang T. Metagenomic and metatranscriptomic analysis of microbial community structure and gene expression of activated sludge. *PLoS One* 2012;15:e38183. 10.1371/journal.pone.0243233 PMC3364235 22666477

[65] Belasco JG . All things must pass: contrasts and commonalities in eukaryotic and bacterial mRNA decay. *Nat Rev Mol Cell Biol* 2010;11:467–78. 10.1038/nrm2917 20520623 PMC3145457

[66] Rauhut R, Klug G. mRNA degradation in bacteria. *FEMS Microbiol Rev* 1999;23:353–70. 10.1111/j.1574-6976.1999.tb00404.x 10371038

[67] Deutscher MP . Degradation of RNA in bacteria: comparison of mRNA and stable RNA. *Nucleic Acids Res* 2006;34:659–66. 10.1093/nar/gkj472 16452296 PMC1360286

[68] Kostygov AY, Karnkowska A, Votýpka J. et al. Euglenozoa: taxonomy, diversity and ecology, symbioses and viruses. *Open Biol* 2021;11:200407. 10.1098/rsob.200407 33715388 PMC8061765

[69] Abinandan S, Shanthakumar S. Challenges and opportunities in application of microalgae (*Chlorophyta*) for wastewater treatment: a review. *Renew Sust Energ Rev* 2015;52:123–32. 10.1016/j.rser.2015.07.086

[70] Gubbels MJ, Duraisingh MT. Evolution of apicomplexan secretory organelles. *Int J Parasitol* 2012;42:1071–81. 10.1016/j.ijpara.2012.09.009 23068912 PMC3583008

