# Supplementary material for: Longitudinal dynamics and cross-domain interactions of eukaryotic populations in wastewater treatment plants
Source: ISME J. 2025 Apr 4;19(1):wraf058. doi: 10.1093/ismejo/wraf058 (PMC12021597; doi:10.1093/ismejo/wraf058)
Supplement: EUK_MS_SI_V10_wraf058 [file euk_ms_si_v10_wraf058.pdf]

Supplementary Materials for

Longitudinal dynamics and cross-domain interactions of eukaryotic populations in wastewater treatment plants

Yue Huang[1], Xuemei Mao[1], Xiawan Zheng[1], Yuxiang Zhao[1], Dou Wang[1], Mengying Wang[1], Yiqiang Chen[1], Lei Liu[1], Yulin Wang[1], Martin F. Polz[2], Tong Zhang[1,3,4,5,6,*]

[1] Environmental Microbiome Engineering and Biotechnology Laboratory, Center for Environmental Engineering Research, Department of Civil Engineering, The University of Hong Kong, Hong Kong SAR, China

[2] Division of Microbial Ecology, Centre for Microbiology and Environmental Systems Science, University of Vienna, Vienna, 1030, Austria.

[3] School of Public Health, The University of Hong Kong, Hong Kong SAR, China

[4] Macau Institute for Applied Research in Medicine and Health, Macau University of Science and Technology, Macao SAR, China

[5] State Key Laboratory of Marine Pollution, City University of Hong Kong, Hong Kong SAR, China

[6] Shenzhen Innovation and Research Institute, The University of Hong Kong, Shenzhen, China

*Corresponding author.

Email: zhangt@hku.hk (T. Zhang); Mailing address: Room 6-31, Haking Wong Building, Department of Civil Engineering, The University of Hong Kong, Pokfulam Road, Hong Kong SAR 999077, China; Tel.: +852-28578551; Fax: +852-25595337.

List of Figures

Figure S1. Gene annotation of the predicted proteins based on eggnog database 6.0.

Figure S2. Phylogenetic distribution of eukaryotic ASVs extracted from 143 activated sludge samples.

Figure S3. Features of prokaryotic MAGs. Genome size, GC content, and coding density for prokaryotic families.

Figure S4. Dynamics of microbial communities at the domain level.

Figure S5. Variation of the top 20 most abundant eukaryotic phyla before and after the bleaching event.

Figure S6. Monthly and seasonal dynamics of eukaryotes in activated sludge over 13 years.

Figure S7. Temporal dynamics of 1109 dereplicated prokaryotic MAGs at the phylum level.

Figure S8. Alpha-diversity distribution of the eukaryotic and prokaryotic communities in activated sludge system.

Figure S9. Seasonal alpha-diversity distribution of the eukaryotic and prokaryotic communities in activated sludge system.

Figure S10. Distance-based redundancy analysis of the samples with environmental and operational variables.

Figure S11. Operation parameters over 13 years.

Figure S12. Pairwise interaction strength among microbes in activated sludge at the phylum level.

Figure S13. The expression level of microbial populations in activated sludge.

Figure S14. Temporal transcriptional dynamics of dominant eukaryotic phyla in ST WWTPs over 1 year.

Figure S15. Spatial diversity of microbial communities in six local WWTPs.

Figure S16. Rarefaction curve of detected eukaryotic ASVs.

List of Tables

Table S1. Wastewater treatment plants (WWTPs) under study.

Table S2. Detailed information on all activated sludge samples.

Table S3. Summary of collected operational parameters in the Sha Tin WWTPs.

Table S4. Taxonomy annotation of retrieved 18S rRNA genes.

Table S5. Features of prokaryotic metagenome-assembled genomes (MAGs).

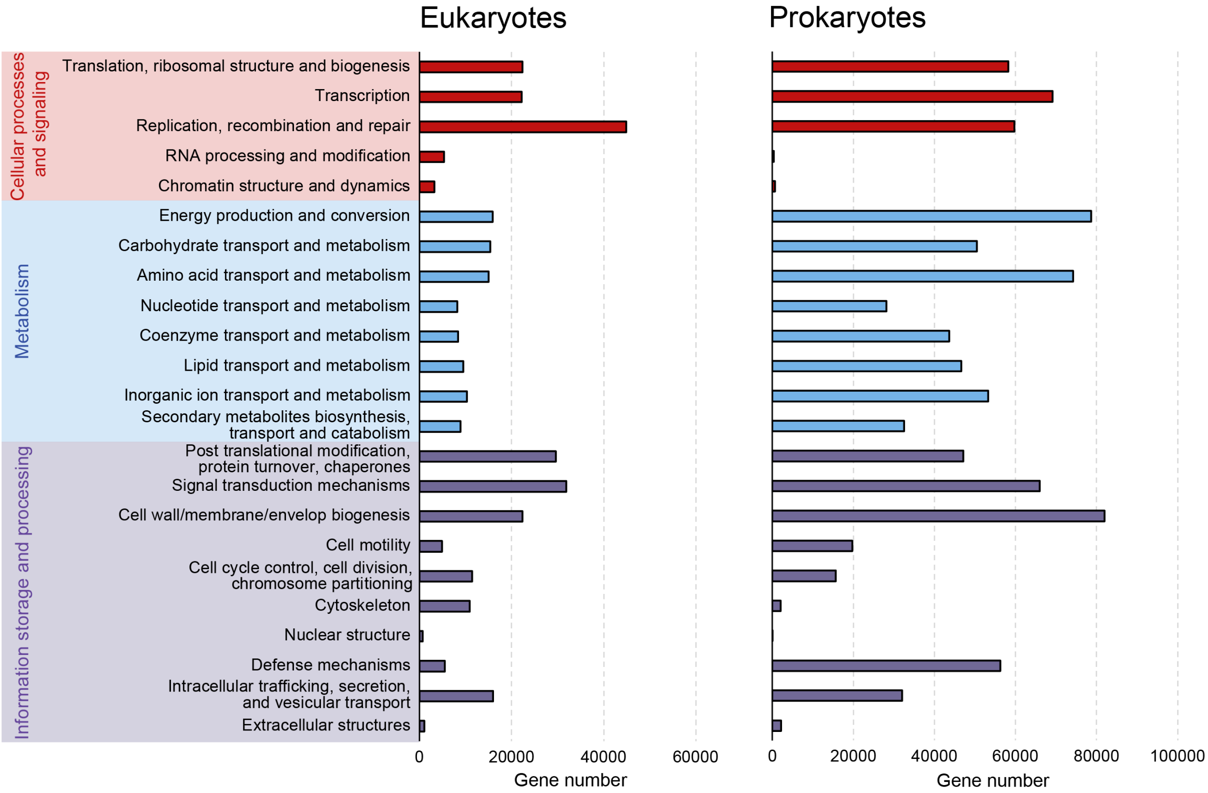

Figure S1. Gene annotation of the predicted proteins based on eggnog database 6.0.

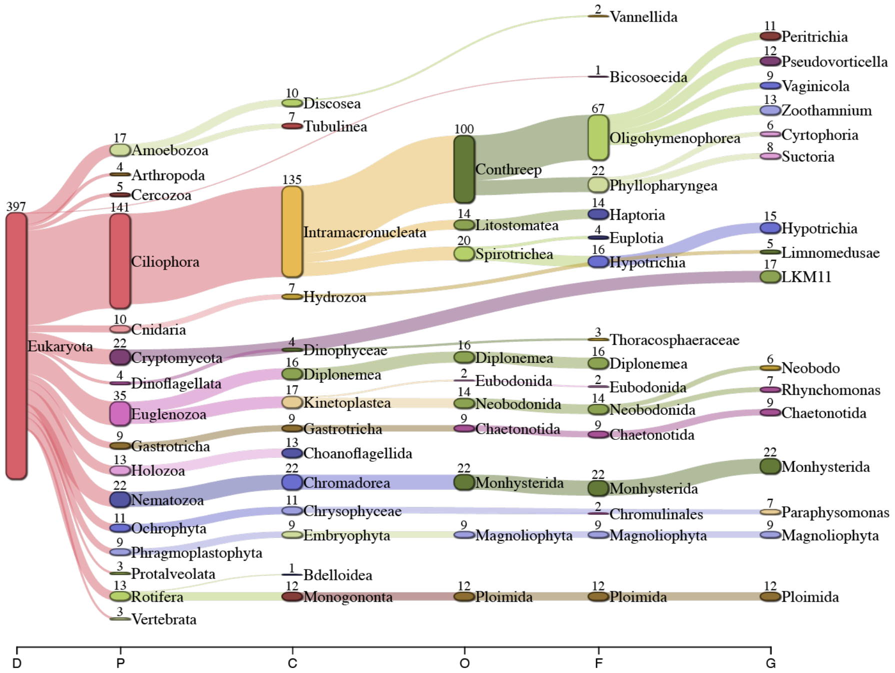

Figure S2. Phylogenetic distribution of eukaryotic ASVs extracted from 143 activated sludge samples. The taxonomy was assigned by QIIME2 (v2024.5) based on the SILVA SSU database release 138, and the Sankey diagram was created with Pavian (v1.0).

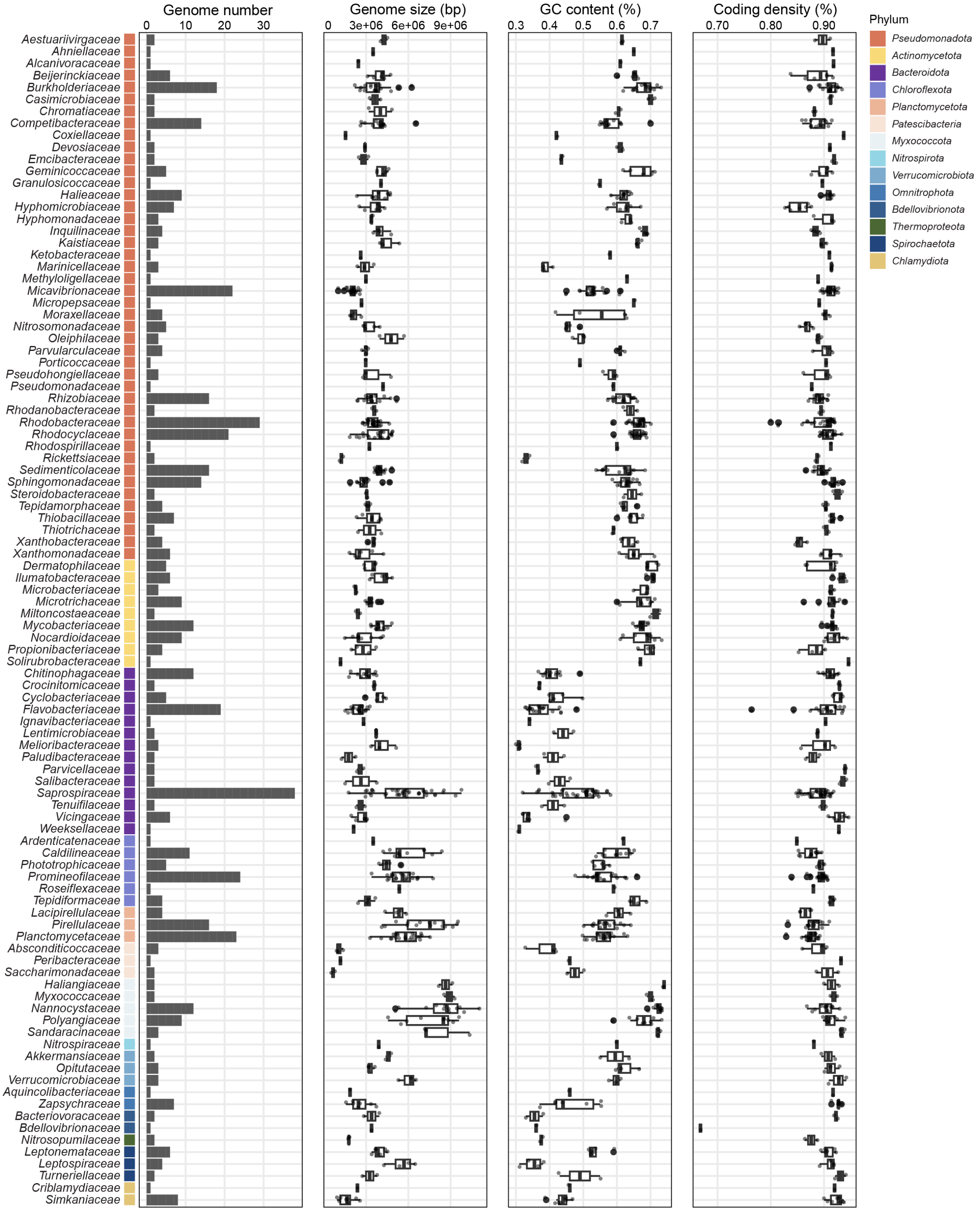

Figure S3. Features of prokaryotic MAGs. Genome size, GC content, and coding density for prokaryotic families, colored by phylum. In total, 1109 MAGs were retrieved in this study, but only 572 were annotated at the family level as shown in the figure. Detailed information on all MAGs was summarised in Table S5.

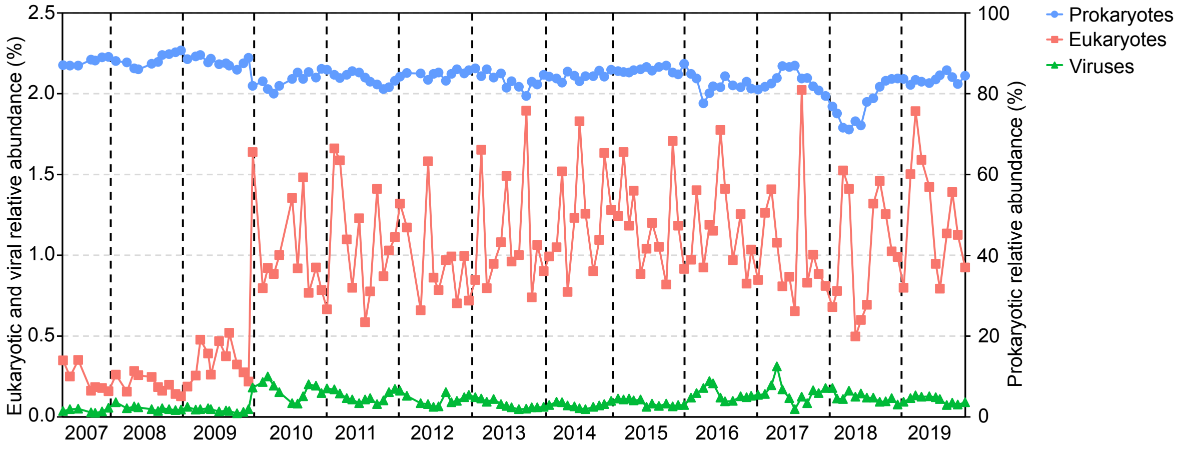

Figure S4. Dynamics of microbial communities at the domain level. The viral contigs were identified using geNomad (v1.7.4) with the "genomad end-to-end" workflow. The sequences shorter than 5000 bp with a virus score lower than 0.9 were removed from the identified viral contigs.

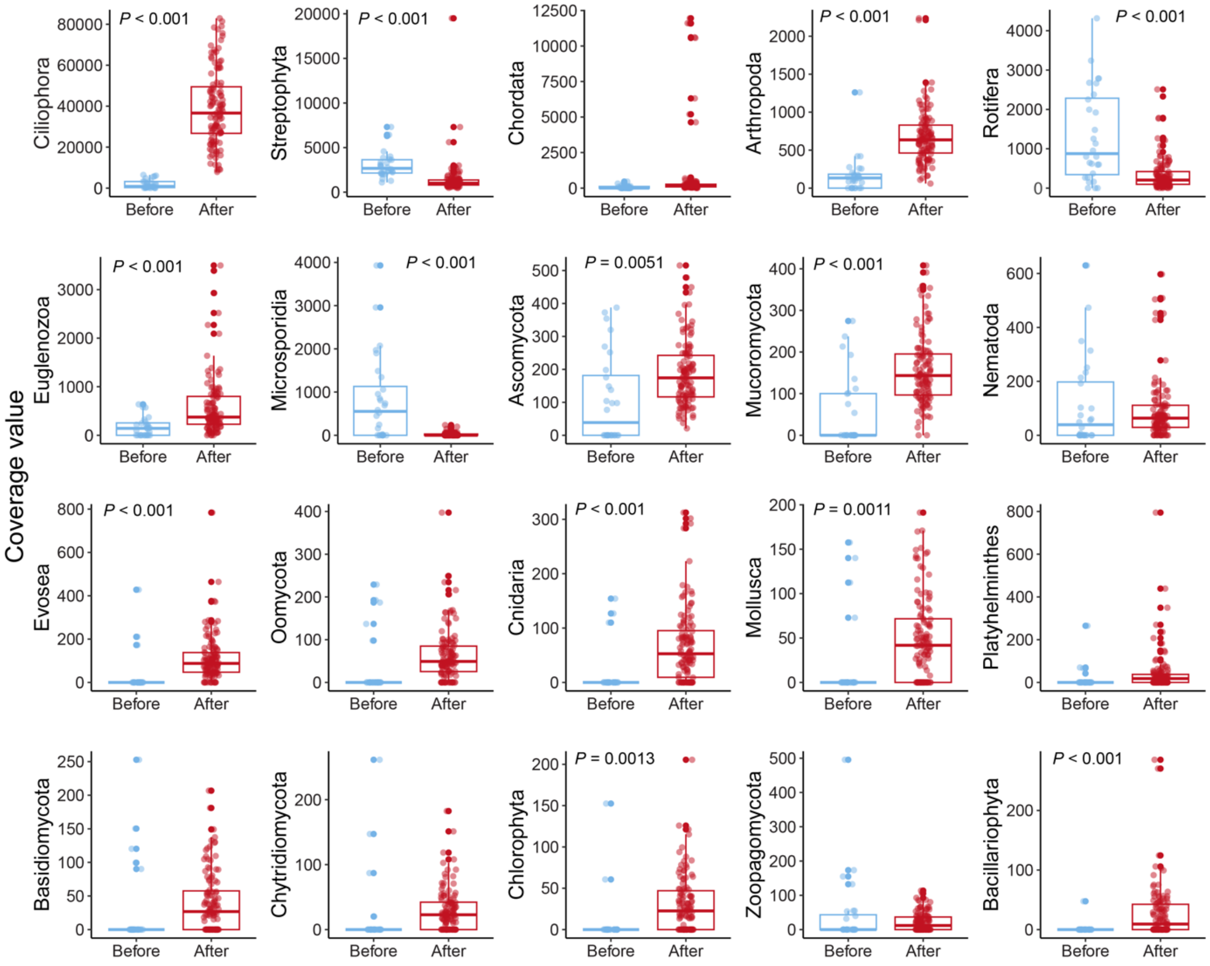

Figure S5. Variation of the top 20 most abundant eukaryotic phyla before and after the bleaching event. The taxonomy annotation was performed using MetaEuk.

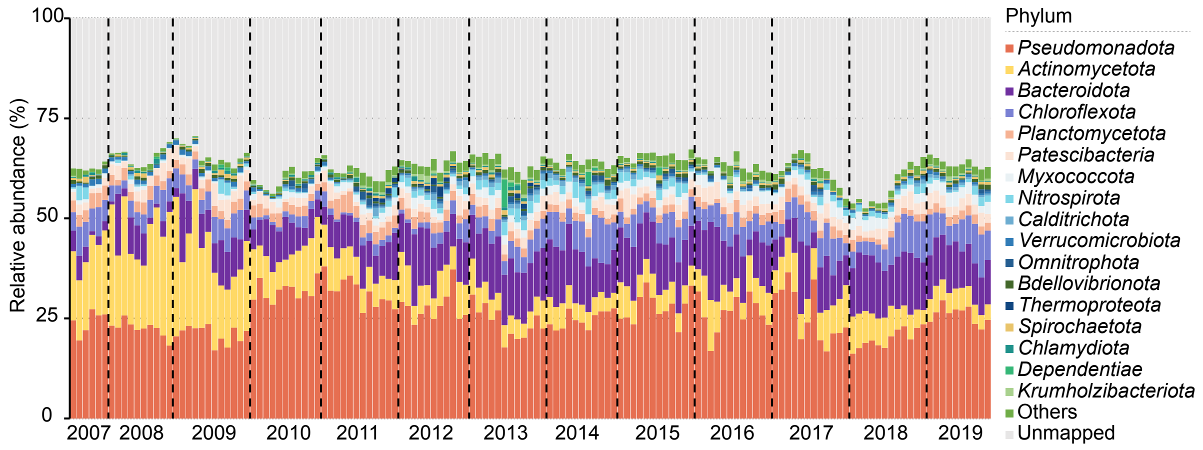

Figure S6. Temporal dynamics of 1109 dereplicated prokaryotic MAGs at the phylum level. Accumulative relative abundance was calculated based on the ratio of recruited reads of the MAGs.

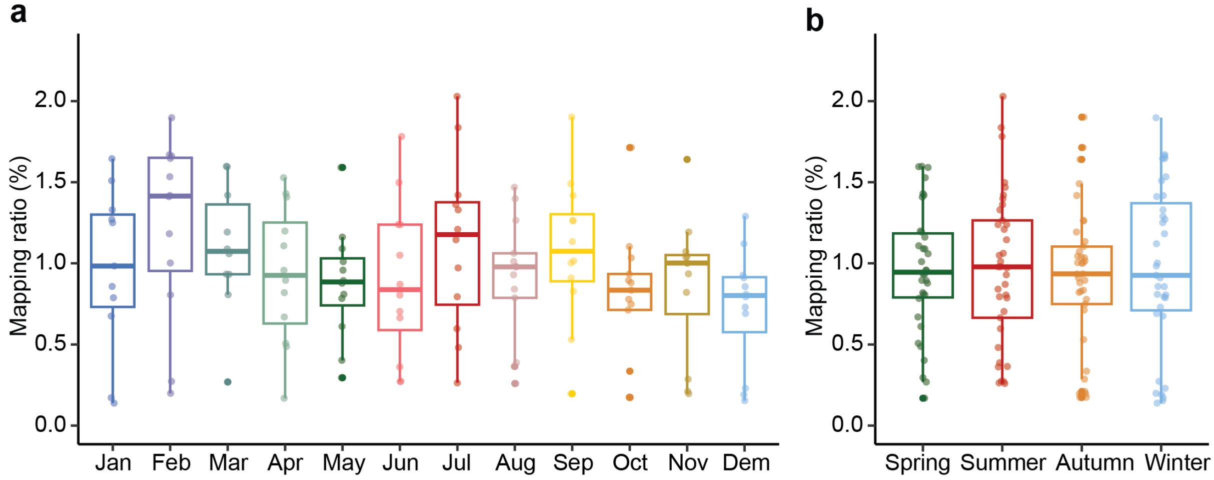

Figure S7. Monthly (a) and seasonal (b) dynamics of eukaryotes in activated sludge over 13 years. The mapping ratio was calculated based on the ratio of recruited reads of the eukaryotic contigs.

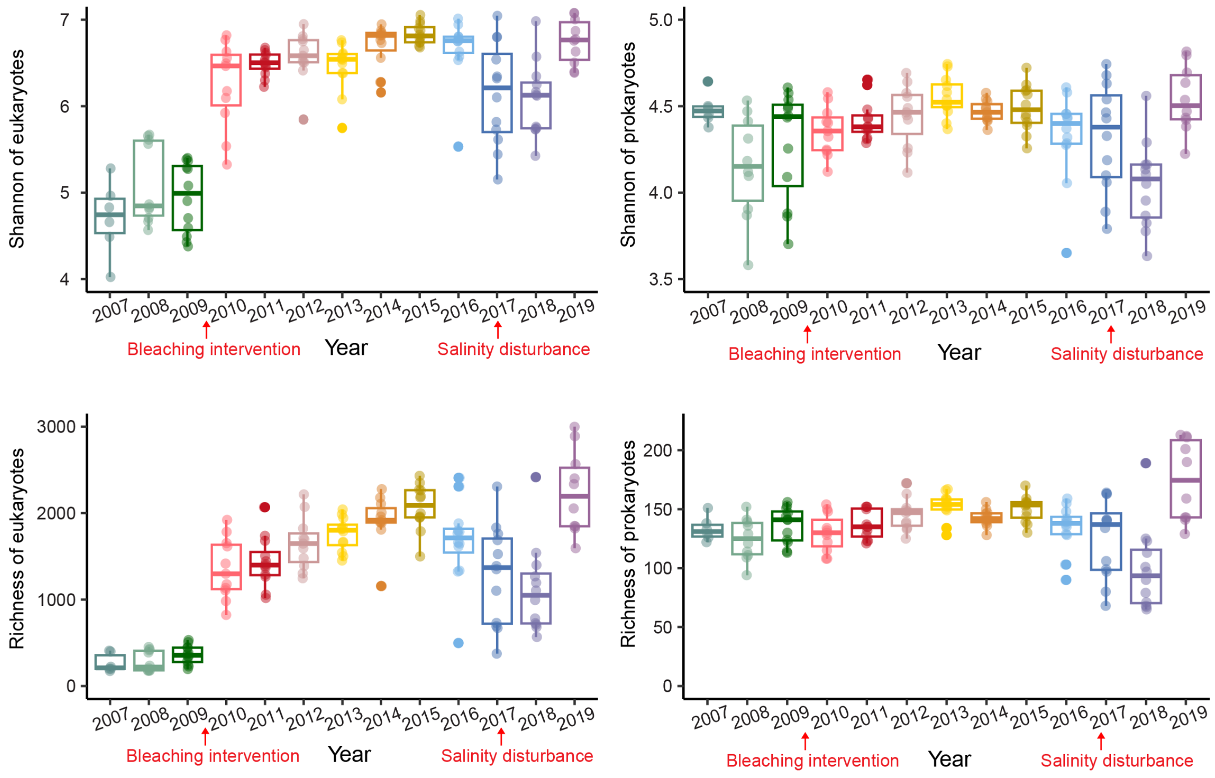

Figure S8. Alpha-diversity distribution of the eukaryotic and prokaryotic communities in activated sludge system.

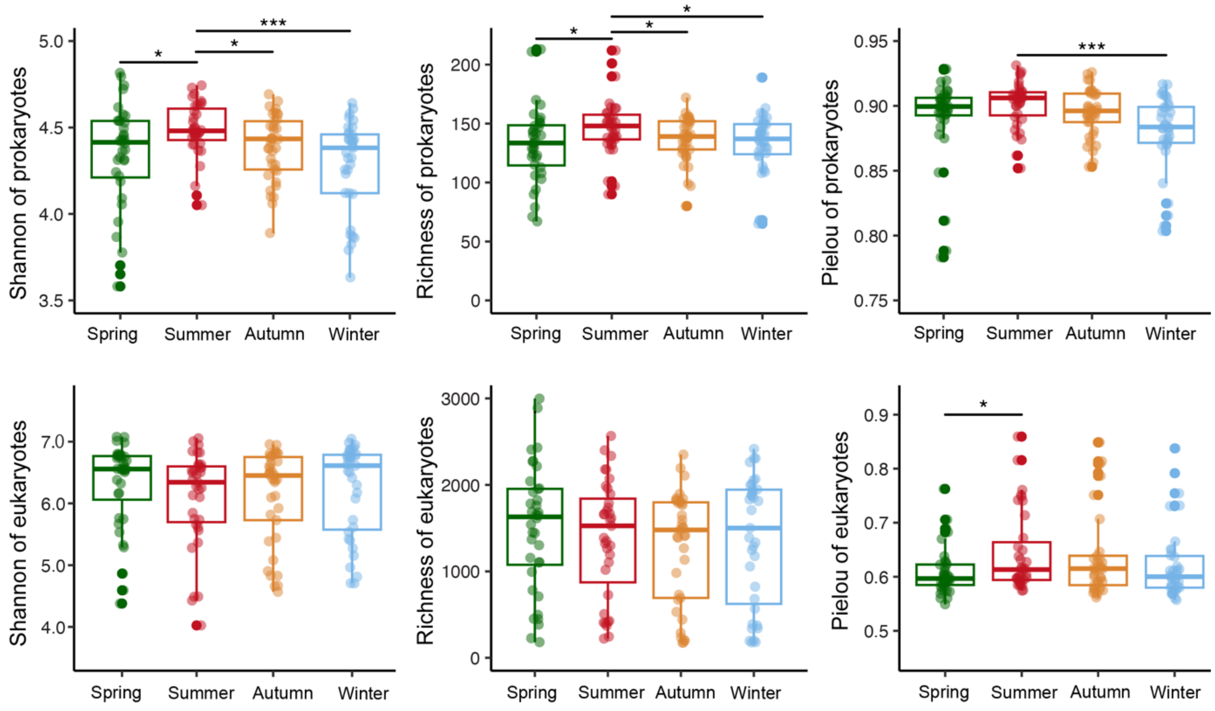

Figure S9. Seasonal alpha-diversity distribution of the eukaryotic and prokaryotic communities in activated sludge system.

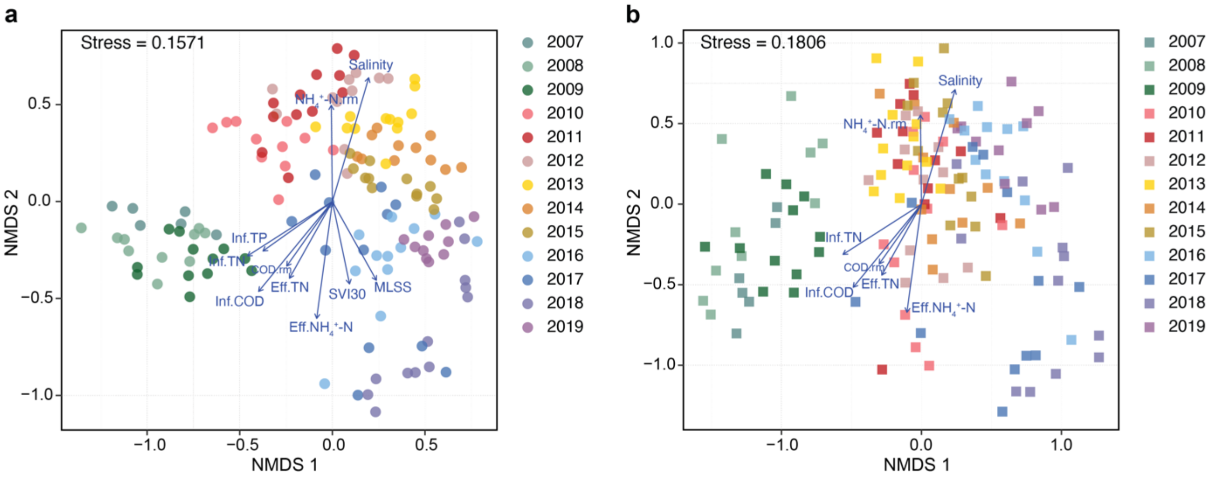

Figure S10. Prokaryotic (a) and eukaryotic (b) distance-based redundancy analysis of the samples with environmental and operational variables. Activated sludge samples were colored by year. The result *p* values underwent false discovery correction with the Bonferroni procedure. Only the variables with *P* value < 0.05 were shown in the figure. Inf.: influent; Eff.: effluent; TN.: total nitrogen; TP: total phosphorous; COD: Chemical oxygen demand; rm: removal rate; MLSS: mixed liquor suspended solids; SVI30: sludge volume index at 30 min

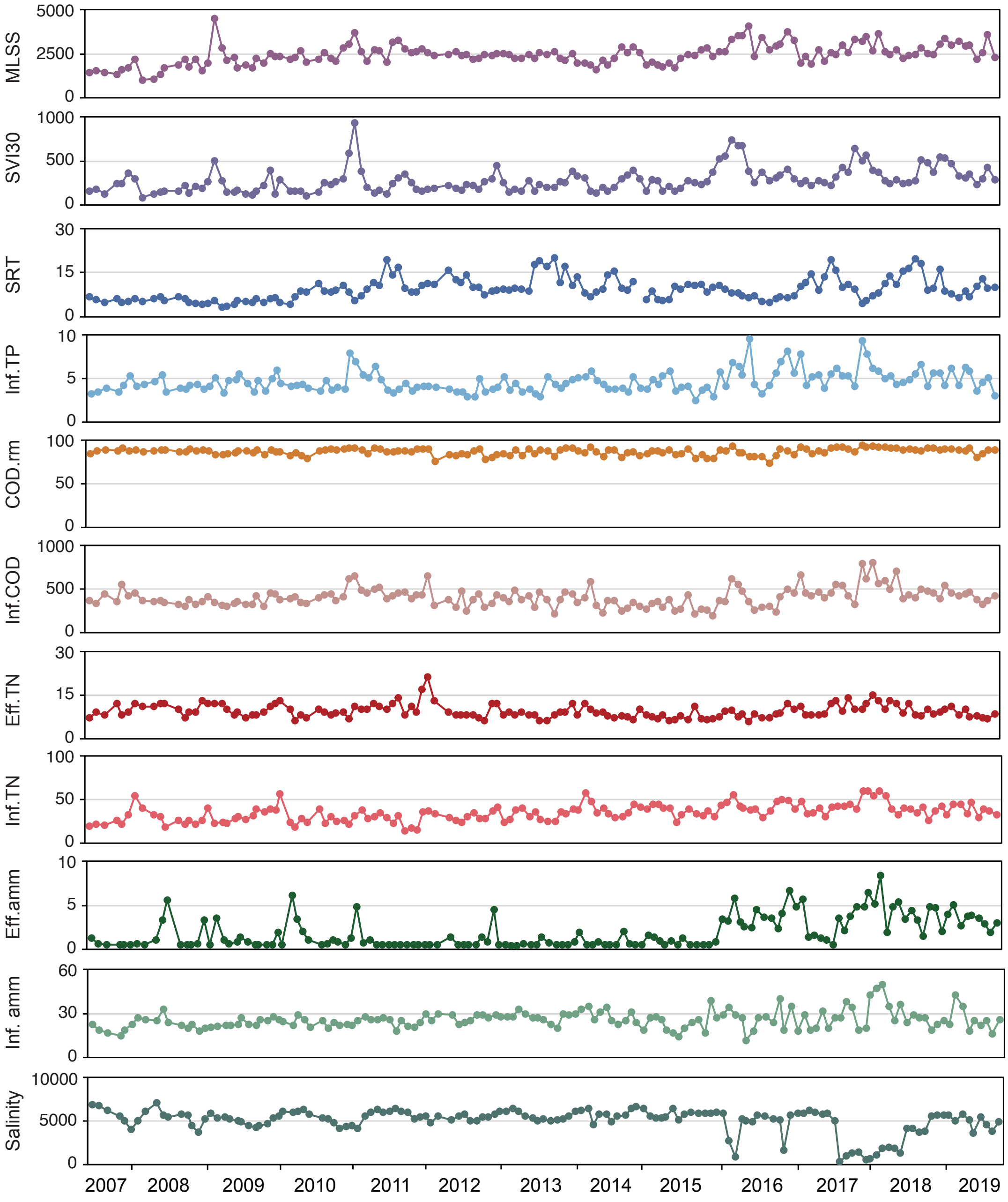

Figure S11. Operation parameters over 13 years, including salinity, influent and effluent ammonia nitrogen concentrations, influent and effluent total nitrogen concentrations, influent chemical oxygen demand (COD) concentration, COD removal efficiency, influent total phosphate, SVI30, and mixed liquor suspended solids (MLSS). The salinity of the sewage was measured by mg/L Cl^-.

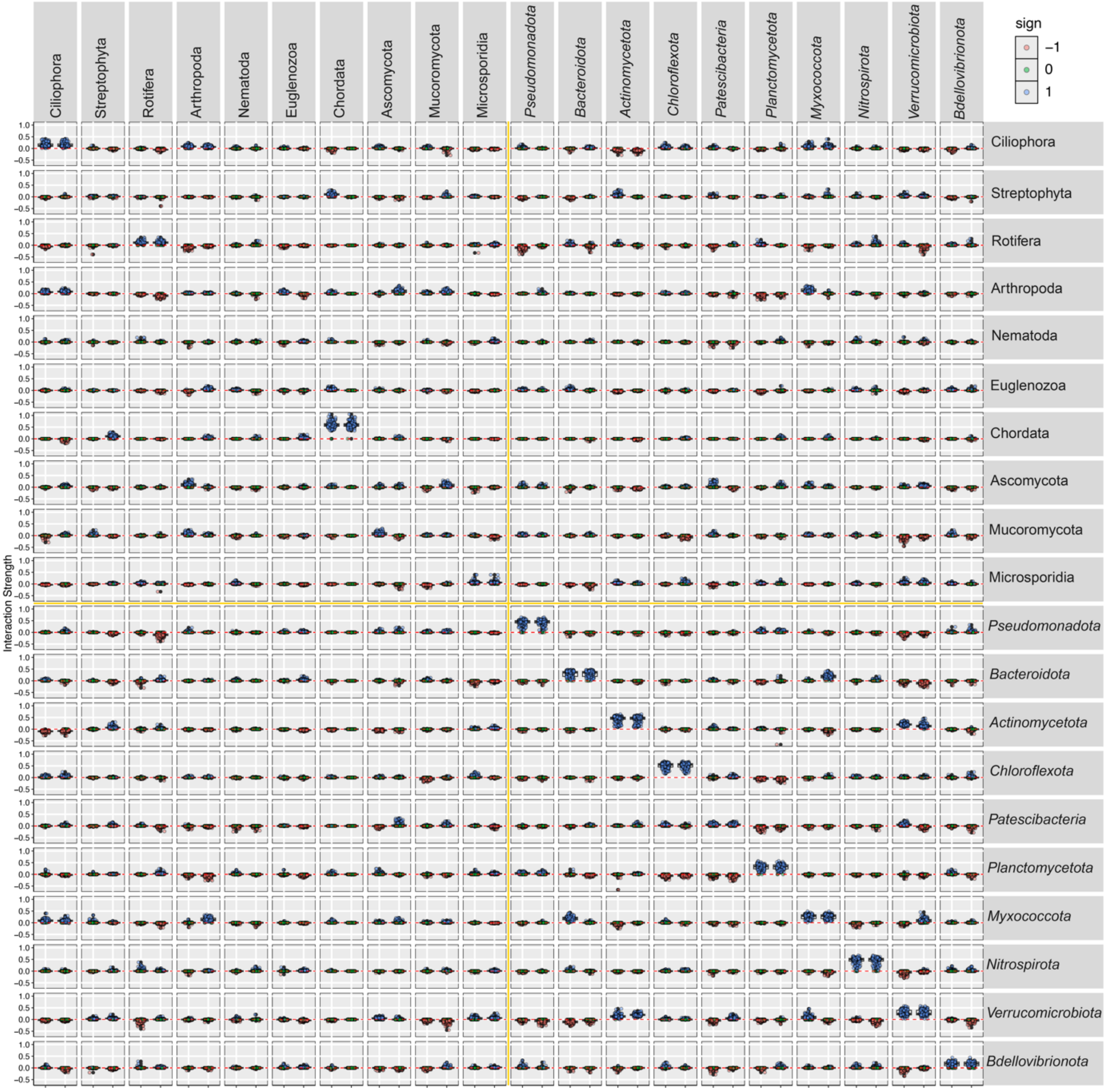

Figure S12. Pairwise interaction strength among microbes in activated sludge at the phylum level. The Jacobian elements were inferred by the regularized S-map. The positive and negative strength was distinguished by blue and red colors, respectively. The left boxplot indicates the column-to-row interaction strength, while the right boxplot displays the row-to-column interaction strength. The ecological relationships include cooperation (+/+), commensalism (+/0), predation (+/–), amensalism (–/0), competition (–/–), and no interaction (0/0).

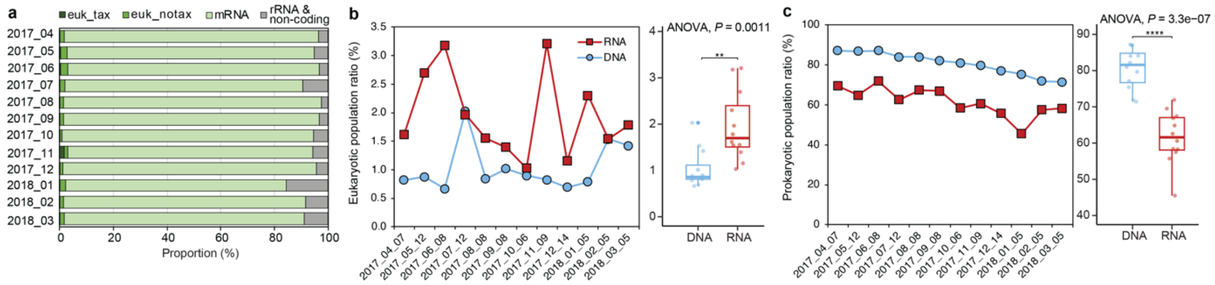

Figure S13. The expression level of microbial populations in activated sludge. (a) The proportion of non-coding rRNA (6.30 □ 3.76%) and mRNA (93.70 □ 3.76%) sequences in transcriptional data. Of the identified eukaryotic reads (1.95 □0.74%), only ~10.7% could be taxonomically assigned. tax: taxonomy. The expression level of eukaryotic (b) and prokaryotic (c) populations.

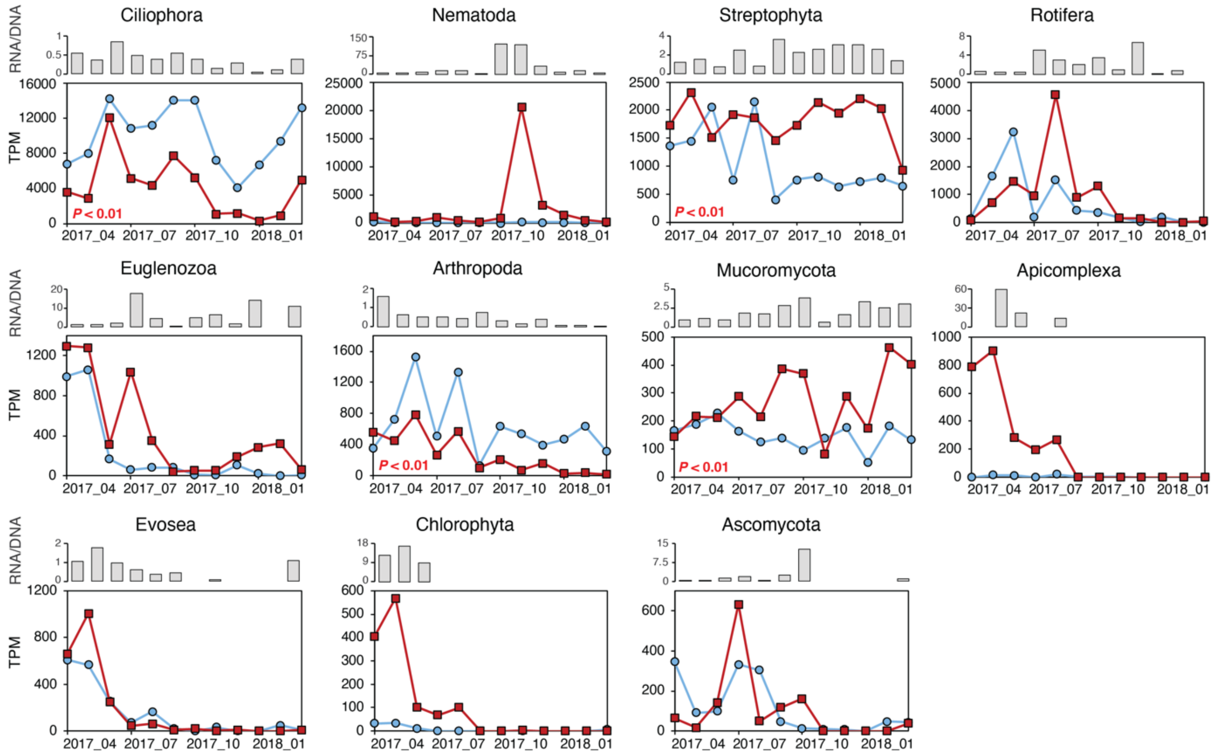

Figure S14. Temporal transcriptional dynamics of dominant eukaryotic phyla (with average coverage value over 100 in metagenomic or metatranscriptomic data) in ST WWTPs over 1 year.

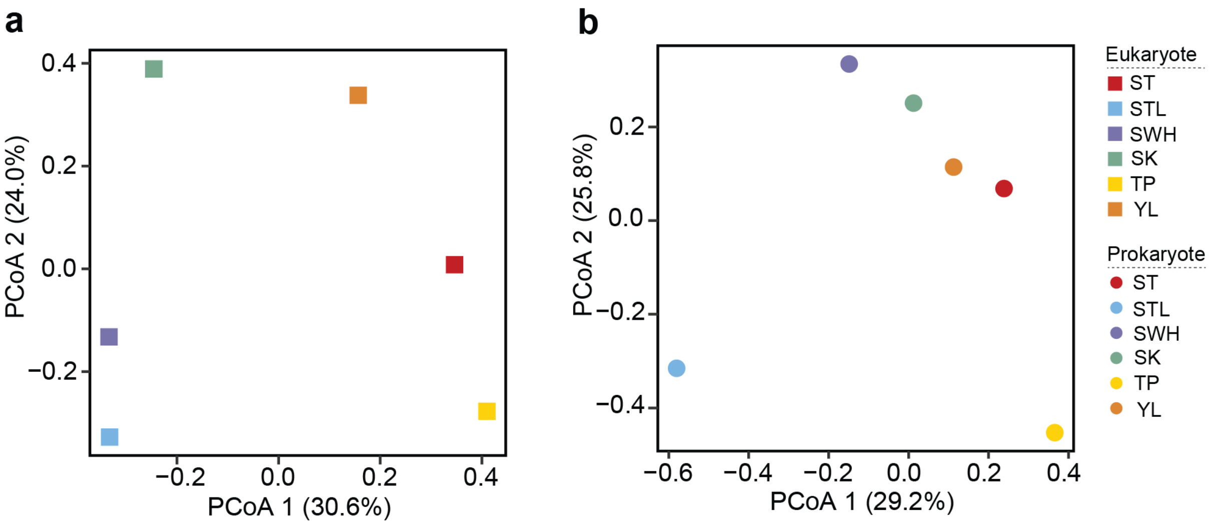

Figure S15. Spatial diversity of microbial communities in six local WWTPs. The PCoA plot of six local WWTPs based on Bray–Curtis distances of eukaryotic (a) and prokaryotic (b) populations.

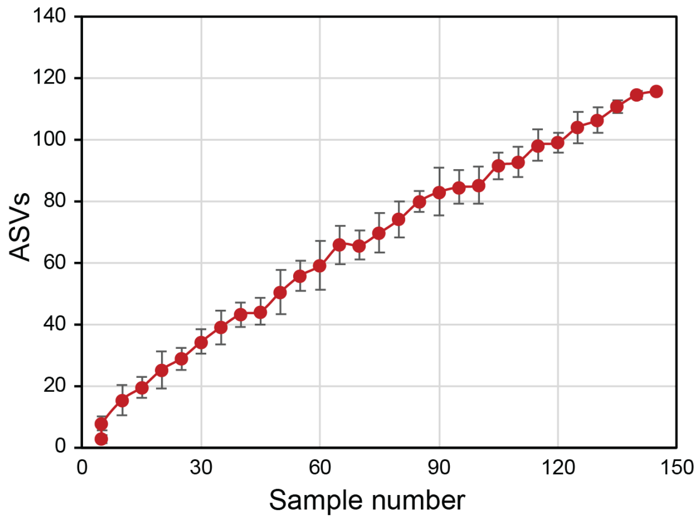

Figure S16. Rarefaction curve of detected eukaryotic ASVs based on 10 permutations.
